# Oxysterols from a Marine Sponge *Inflatella* sp. and Their Action in 6-Hydroxydopamine-Induced Cell Model of Parkinson’s Disease

**DOI:** 10.3390/md16110458

**Published:** 2018-11-21

**Authors:** Sophia A. Kolesnikova, Ekaterina G. Lyakhova, Anatoly I. Kalinovsky, Roman S. Popov, Ekaterina A. Yurchenko, Valentin A. Stonik

**Affiliations:** 1 G.B. Elyakov Pacific Institute of Bioorganic Chemistry (PIBOC), Prospect 100-let Vladivostoku 159, Vladivostok 690022, Russia; elyakhova@inbox.ru (E.G.L.); kaaniv@piboc.dvo.ru (A.I.K.); prs_90@mail.ru (R.S.P.); dminae@mail.ru (E.A.Y.); office@piboc.dvo.ru or schoolNS@dvfu.ru or stonik@piboc.dvo.ru (V.A.S.); 2 School of Natural Science, Far Eastern Federal University, Sukhanova St., 8, Vladivostok 690000, Russia

**Keywords:** secondary metabolites, oxygenated steroids, marine sponge, *Inflatella*, NMR, ROS, Parkinson’s disease, structure-activity relationship

## Abstract

Four new oxysterols **1**–**4** along with previously known oxygenated sterols **5**–**14** were isolated from the sponge *Inflatella* sp., collected from the Sea of Okhotsk. Structures of **1**–**4** were elucidated by the detailed NMR spectroscopic and mass-spectrometric analyses as well as by comparison of the corresponding experimental data with those reported in literature. The influence of compounds **1**–**14** on the viability of neuronal Neuro2a cells treated by 6-hydroxydopamine and reactive oxygen species (ROS) formation in these cells was investigated.

## 1. Introduction

Oxysterols, formed either enzymatically or by auto-oxidation, have a second oxygen function in addition to that of C-3 when compared with natural sterols. These compounds are of particular interest due to important biological functions of some of them and various bioactivities, including effects on lipid metabolism, platelet aggregation, apoptosis, different receptors and proteins [1]. Generally, oxysterols attract a great attention by the contribution to many physiological processes, including those connected with pathophysiology of the neurodegenerative diseases [2]. As a key trend, the roles of two brain sterols, 24*S*-hydroxycholesterol and 27-hydroxycholesterol, have been studied for years and discussed in multiple reports [3,4,5]. Among others, 24*S*-hydroxycholesterol has been reported to be markedly reduced in the circulation of patients with Parkinson’s disease (PD) [4]. The treatment with 27-hydroxycholesterol or a combination of 24*S*-hydroxycholesterol and 27-hydroxycholesterol reduced the levels of noradrenaline, whereas the treatment with 24*S*-hydroxycholesterol alone had no effect. With these results, the authors demonstrated that oxysterols could regulate the proteins involved in the development of PD [5].

Recently, some other oxysterols were found to be exported from the brain: 7β-hydroxycholesterol, 7-ketocholesterol, 3β,5α-dihydroxycholestan-6-one, 7α-hydroxy-3-oxocholest-4-enoic acid, 7α,25-dihydroxycholest-4-en-3-one, and (25*R*)-7α,26-dihydroxycholest-4-en-3-one. It was underlined that these transfers were observed when total oxysterols were measured [6,7]. These facts give a new quest to disclose possible roles and bioactivities of non-common oxysterols, including those of marine origin, in development of PD and other neurodegenerative diseases.

Recent studies highlighted the point that even well-known oxygenated sterols might be a critical source of new medicine leads. Thus, hecogenin and cholest-4-en-3-one showed significant inhibitory activity (EC_50_ values of 116.3 and 390.6 µM, respectively) against the human β-site amyloid cleaving enzyme (BACE1), which has been considered as an effective drug target for treatment of Alzheimer’s disease [8].

The search for new oxysterols in marine organisms and the studies on their action in model cell systems could be also considered as an approach to the creation of new pharmaceutical leads against neurodegenerative diseases. As a part of our chemical investigations of steroidal compounds from marine sponges [9,10,11], we have studied oxysterols of a cold-water marine sponge *Inflatella* sp. The sponges belonging to the genus *Inflatella* are insufficiently studied for their secondary metabolites, though some bioactivities of their extracts were noted [12]. The ethanol extract of the studied *Inflatella* sp. sample was preliminary analyzed by thin-layer chromatography during the screening of sponge samples from PIBOC collection. The results revealed that in contrast with extracts of other sponges, it contained the oxygenated steroids visually detected on chromatograms as a number of bright colored spots (Figure S1). Herein we describe the isolation and structural elucidation of fourteen individual oxysterols of *Inflatella* sp., including four new compounds as well as the results of their biotesting using a cell model of PD. Part of this work has been presented at the 3rd International Symposium on Life Sciences, Vladivostok, Russia, September 2018.

## 2. Results and Discussion

**Figure 1:**
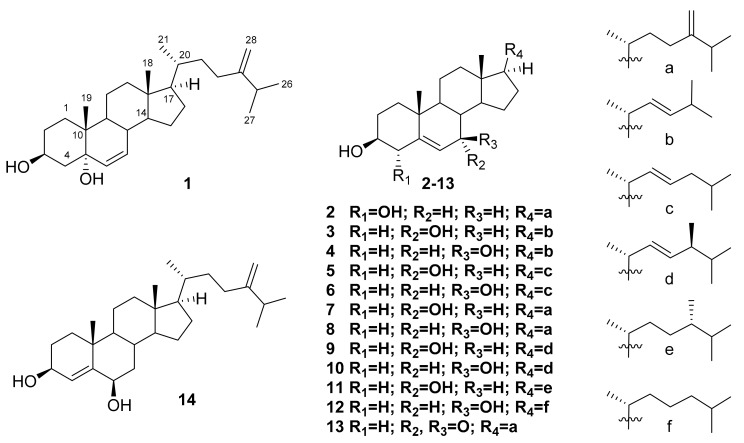
Structures of compounds **1**–**14**.

The ethanol extract of a sponge *Inflatella* sp., collected in the Sea of Okhotsk, was concentrated and partitioned between distilled water and CHCl_3_. The obtained organic layer was further separated using a combination of column chromatography and normal- or reverse-phase HPLC to yield compounds **1**–**14** (Figure 1). To identify the structures of isolated compounds, along with the absolute stereochemistry, their NMR and HRESI MS characteristics were analyzed and compared with previously published data [13,14,15,16,17,18]. 

Compound **1** was isolated as white amorphous powder with molecular formula C_28_H_46_O_2_, established from (+) HRESI MS (*m/z* 437.3393 [M + Na]^+^) and ^13^C NMR spectroscopic data (Figures S2 and S4; Table 1). The ^1^H and ^13^C NMR spectra (Table 1 and Table 2; Figures S3 and S4) brought out signals characteristics of steroidal derivatives. Namely, the ^1^H NMR (Table 2) and HSQC (Figure S7) spectra allowed to refer signals of five methyl groups as belonging to Me-18 (δ_H_ 0.71, s; δ_C_ 12.4), Me-19 (δ_H_ 0.92, s; δ_C_ 14.9), Me-21 (δ_H_ 0.96, d, *J* = 6.5 Hz; δ_C_ 18.9), Me-26 (δ_H_ 1.03, d, *J* = 6.8 Hz; δ_C_ 22.1), and Me-27 (δ_H_ 1.02, d, *J* = 6.8 Hz; δ_C_ 22.2). The chemical shifts at δ_H_ 4.12, m; δ_C_ 67.4, (HC-3), at δ_H_ 5.57, dd, *J* = 9.8, 2.7 Hz; δ_C_ 133.5, (HC-6) and at δ_H_ 5.63, dd, *J* = 9.8, 1.6 Hz; δ_C_ 133.4 (HC-7) along with oxygenated non-protonated carbon signal δ_C_ 74.2 (C-5), suggested the 3β,5α-6-ene steroidal core [13,14]. It was supported by close similarity of the corresponding ^13^C NMR signals of **1** and known cholest-6-ene-3β,5α-diol while the epimoric compound with 3β,5β-6-ene core was quite different [13]. As the signals at δ_H_ 4.66 (s) and 4.72 (s) showed an exo-methylene double bond (δ_C_ 106.3 and 157.1), the 24(28)-ergostene side-chain was deduced for compound **1**. Therefore, the structure of new compound **1** was established as 24-methylcholesta-6,24(28)-diene-3β,5α-diol.

Compound **2** has the molecular formula C_28_H_46_O_2_ that was determined by (+) HRESI MS *m/z* 437.3393 [M + Na]^+^ and ^13^C NMR (Figures S2 and S9; Table 1). The NMR data of **2** (Table 1 and Table 2; Figures S8–S11) also confirmed its steroidal framework. At first, they showed the same type of side-chain in the structure **2** as in **1** by the presence of very close chemical shifts in the spectra of both compounds. The remaining signals were attributed to Me-18 (δ_H_ 0.69, s; δ_C_ 11.9), Me-19 (δ_H_ 1.03, s; δ_C_ 20.2), 4α-OH (δ_H_ 4.06, m; δ_C_ 75.2), and trisubstituted 5(6)-double bond (δ_H_ 5.74, dt, *J* = 5.5, 2.2 Hz; δ_C_ 117.8, HC-6 and δ_C_ 142.2, C-5) of the previously described 4α-hydroxylated tetracyclic system. Really, the both 4α-hydroxy- and 4β-hydroxycholesterols were obtained and well characterized during the investigation of cholesterol autoxidation [15]. As a result of the thorough comparison of 1D and 2D NMR experimental data of **2** with published values, the structure of this new oxysterol was deduced as 24-methylcholesta-5,24(28)-diene-3β,4α-diol.

The structure of the compound **3** corresponds to the molecular formula C_26_H_42_O_2_ (HRESI MS *m/z* 409.3081 [M + Na]^+^) (Figures S2 and S14; Table 1). It has a common ∆^5^-3β,7α-diol steroidal core that has been confirmed by the signals of Me-18 (δ_H_ 0.70, s; δ_C_ 11.9), Me-19 (δ_H_ 1.00, s; δ_C_ 18.2), two methines, connected with hydroxy groups (δ_H_ 3.59, m; δ_C_ 71.4, HC-3 and δ_H_ 3.85, br s; δ_C_ 65.3, HC-7), and the 5(6)-double bond (δ_H_ 5.60, dd, *J* = 5.3, 1.6 Hz; δ_C_ 123.9, HC-6 and δ_C_ 146.2, C-5) [16]. However, the molecular formula and characteristic signals of the 22*E*-double bond (δ_H_ 5.18, dd, *J* = 15.3, 6.6 Hz; δ_C_ 133.6, HC-22 and δ_H_ 5.27, dd, *J* = 15.3, 6.6 Hz; δ_C_ 134.9, HC-23), as well as Me-26,27 signals (δ_H_ 0.94, d, *J* = 6.7 Hz; 6H; δ_C_ 22.7) showed that this new steroid was characterized by the presence of the 22*E*-unsaturated 24-*nor*-side chain [17] (Table 1 and Table 2; Figures S13–S18). Finally, the structure of new oxysterol **3** was determined as (22*E*)-24-*nor*-cholesta-5,22-diene-3β,7α-diol (**3**) that was also in good agreement with ROESY and HMBC data (Figures S16 and S18).

The C_26_H_42_O_2_ molecular formula ((+) HRESI MS *m/z* 409.3081 [M + Na]^+^) of compound **4** together with similar NMR spectral characteristics (Table 1 and Table 2; Figures S2, S19–S24) suggested the presence of a side-chain the same as in compound **3**. The core parts of these compounds also demonstrated a set of close ^1^H NMR signals except for the chemical shift values, constants, and multiplicity of H-6 (δ_H_ 5.29, t, *J* = 2.2 Hz) and H-7 (δ_H_ 3.85, dt, *J* = 8.3, 2.2 Hz). Both mentioned signals were similar to those described previously [16,18] and suggested the 7β-OH stereochemistry in **4**. All these data along with confirmation by ROESY and HMBC spectra (Figures S22 and S24) allowed to establish the (22*E*)-24-*nor*-cholesta-5,22-diene-3β,7β-diol (**4**) structure of this new oxysterol.

Additionally, the remaining ten isolated compounds **5**–**14** were identified as known oxygenated steroidal derivatives by spectroscopic methods and comparison with reported data including NMR spectra [16,18,19,20]. Most of them were described as naturally occurring metabolites possessing diverse biological activities. Thus, (22*E*)-cholesta-5,22-diene-3β,7α-diol (**5**) was originally isolated from the marine sponge *Cliona copiosa* [16], and demonstrated anti-inflammatory, analgesic and gastroprotective activities as a component of ethanolic fraction of gorgonian *Eunicella singularis* [21]. The (22*E*)-cholesta-5,22-diene-3β,7β-diol (**6**) [16] deterred starfish predators [22]. Selective activity towards DNA repair-deficient yeast mutants and cytotoxicity towards wild-type P-388 murine leukemia cells [23] were showed for 24-methylene-5-cholestene-3β,7α-diol (**7**) [18]. Moreover, the compound **7** was noted as a potential drug development candidate for Alzheimer’s disease due to inhibitory potential against butyrylcholinesterase (BuChE) with IC_50_ 9.5 μM [24]. The 24-methylene-5-cholestene-3β,7β-diol (**8**) [18] was completely inactive in the screening for DNA-damaging agents in the RAD 52 yeast assay (while its epimer **7** was active with IC_50_ 7 μg/mL), and showed moderate cytotoxic activity (IC_50_ 31 μM) in the Vero cell assay, indicating that 7β-OH compound acted by a different mechanism in comparison with its α-counterpart [25]. Both compounds **7** and **8** were also noted as constituents of the royal jelly of honeybees [26].

To the best of our knowledge there are not any reported bioassay results in relation to (22*E*,24*S*)-24-methylcholesta-5,22-diene-3β,7α-diol (**9**) [16], (22*E*,24*S*)-24-methylcholesta-5,22-diene-3β,7β-diol (**10**) [16,18], and (22*E*,24*R*)-24-methylcholesta-5,22-diene-3β,7α-diol (**11**) [16,19]. 7β-Hydroxycholesterol (**12**) isolated from the Red Sea grass *Thalassodendron ciliatum* displayed an inhibitory activity against breast carcinoma cell line MCF-7 (IC_50_ 18.6 ± 0.72 μM) and liver carcinoma cell line Hep G2 (IC_50_ 25.4 ± 0.38 μM). However, it did not show the anti-inflammatory action on carrageenan-induced rat hind paw edema model [27]. When human THP-1 macrophages were exposed with an atheroma-relevant mixture of 7β-hydroxycholesterol (**12**) and 7-ketocholesterol followed by proteome analysis, the alterations in macrophage proteome were indicated with a significant differential expression of 19 proteins [28].

3β-Hydroxy-24-methylene-5-cholesten-7-one (**13**) [18] exhibited the potent inhibitory activity on the interleukin-6 production, with 54.0% inhibition at 10 μM and IC_50_ 9.4 ± 1.2 μM [29]. 24-Methylenecholest-4-ene-3β,6β-diol (**14**) [20] demonstrated the cytotoxic activity against the leukemia P-388 cell line with an IC_50_ 1 μg/mL [30].

Encouraged with the short literature review presented above, that shows different attractive bioactivities of oxysterols, we have made an attempt to evaluate the action of the isolated compounds **1**–**14** on viability of Neuro2a cells and reactive oxygen species (ROS) formation in these cells. In fact, neuroblastoma cells treated by 6-hydroxydopamine (6-OHDA) are used as a cell model of PD [31].

**Figure 2:**
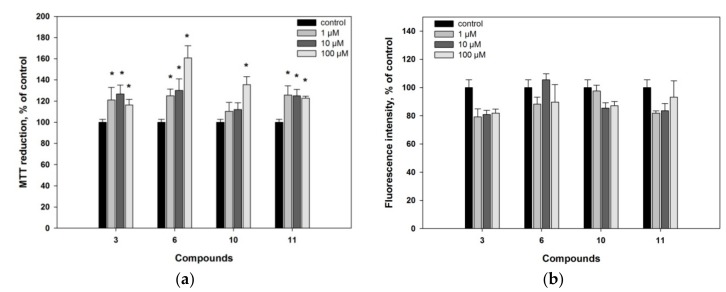
Compounds **3**, **6**, **10** and **11**: (**a**) Caused a statistically significant overestimation of MTT reduction in MTT cell viability assay; (**b**) did not statistically significant affect activity of nonspecific esterase in fluorescein diacetate cell viability test. * Statistically significant differences (*p* ≤ 0.05) between results for control cells and cells incubated with these compounds.

Compounds **4**, **5**, **9** and **12** did not show any notable effects on Neuro2a cell viability. Compounds **1**, **2**, **7**, **8**, **13** and **14** demonstrated slight cytotoxic activity at concentration 100 µM and decreased viability of Neuro2a cells on 25%, 17%, 44%, 27%, 38% and 33%, respectively. It is of interest that all of them have the same structural peculiarity, being the 24 (28)-unsaturated derivatives of ergostane series. No compounds decreasing cell viability more than 50% were found. At concentration of 10 µM, the oxysteroids **1**–**14** were non-toxic against these neuronal cells, and were used in next experiments at the non-toxic concentrations (Figure S25). Moreover, compounds **3**, **6**, **10** and **11** increased the viability of Neuro 2a cells in comparison with non-treated cells, when MTT cell viability test was used (Figure 2a and Figure S25).

Applied MTT assay is one of the most widely exploited approaches in research for measuring cell proliferation, viability and drug cytotoxicity. In living cells, the water-soluble yellow dye MTT is reduced to a dark purple (blue-magenta) colored formazan precipitate, which can be analyzed colorimetrically after dissolving in an organic solvent. It was shown, that the MTT reduction site is not only mitochondria. Non-mitochondrial, cytosolic and microsomal MTT reduction makes the major contribution to an overall reduction. Changes in the activity of dozens of the mitochondrial and non-mitochondrial oxidoreductases, cellular metabolic and energy perturbations, and oxidative stress may significantly impact the MTT assay read out [32]. 

To study action of the tested compounds on cells in details, we additionally used fluorescein diacetate (FDA) assay based on nonspecific esterase activity measuring and thus examined the influence of compounds **3**, **6**, **10** and **11** on proliferation or/and viability of Neuro 2a cells [33]. Compounds **3**, **6**, **10** and **11** did not increase the fluorescence intensity in FDA assay in comparison with control and therefore did not influence significantly on nonspecific esterase activity in Neuro2a cells (Figure 2b). Hence, we could conclude that the observed increasing of MTT reduction was not caused by the influence of tested compounds on cell proliferation.

In fact, the overestimation in MTT assay of the compounds **3**, **6**, **10** and **11** could be caused by alternative metabolic processes. For example, the overestimation reported for rottlerin was explained by dissipation of the inner mitochondrial membrane potential, acceleration of electron transfer and increasing of dehydrogenases activity, oxygen consumption and NADH oxidation [33]. On the other hand the polyphenolic antioxidant resveratrol exhibited increasing of MTT-reducing activity without a corresponding increasing of living cells number [34]. The ability of resveratrol to down-regulate NADPH-oxidase leading to decreased ROS production and thereby provide a protective effect in cardiovascular and neurodegenerative diseases is also well known [35].

As the above reviewed reports described the influence of compounds on intracellular ROS formation, we investigated the effect of compounds **1**–**14** on ROS formation in Neuro 2a cells by short-time 2′,7′-dichlorodihydrofluorescein diacetate (H2DCF-DA) test. Oxysterols **1**–**4**, **8**, **10** and **13** at concentrations of 1 or/and 10 µM slightly decreased the ROS level in Neuro2a cells by 12–16%, while the increasing in ROS formation was not detected for all the studied compounds (Figure S26).

**Figure 3:**
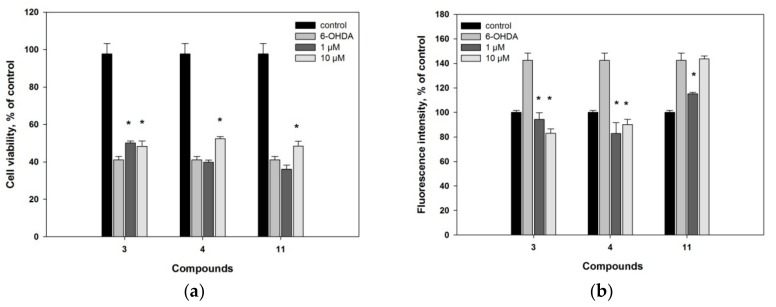
Influence of compounds **3**, **4** and **11** in 6-OHDA-treated Neuro2a cells: (**a**) on cell viability; (**b**) on ROS formation. * Statistically significant differences (*p* ≤ 0.05) between results for 6-OHDA-treated cells and cells incubated with compounds.

PD is the one of the most common age-related motoric neurodegenerative disease, regardless of countries and regions [36]. Pathogenesis of PD includes neuronal death as a result of oxidative stress involved intracellular level of ROS increasing. In this reason, compounds exhibited ROS-scavenger activities could be interesting as neuroprotective agents. All isolated compounds **1**–**14** were studied in 6-OHDA-induced Neuro2a cell model of PD (Figure S26). Only compounds **3**, **4** and **11** affected on viability of 6-OHDA-treated cells (Figure 3a) and ROS formation in these cells (Figure 3b).

As a result, compound **3** increased the viability of 6-OHDA-treated cells by 18% (at the dose of 10 µM) and 22% (1 µM), while compounds **4** and **11** increased cell viability by 28% (10 µM) and 18% (10 µM), correspondingly. All these compounds decreased ROS formation in 6-OHDA-treated cells to normal value in similar manner. Thus, compound **4** exhibits the essential neuroprotective activity in 6-OHDA-induced model of Parkinson’s disease, probably due to ROS scavenging effect. Oxysterols **3**, **6**, **10** and **11** may positively influence on metabolic processes in the Neuro2a cells because they show the overestimation of survival in MTT assay.

## 3. Materials and Methods 

### 3.1. General Methods

Optical rotations were measured on a Perkin-Elmer 343 digital polarimeter (Perkin Elmer, Waltham, MA, USA). The ^1^H and ^13^C NMR spectra were recorded in CDCl_3_ using Bruker Avance III 500 (Bruker BioSpin GmbH, Rheinstetten, Germany) (500.13/125.77 MHz) or Avance III 700 Bruker FT-NMR (Bruker BioSpin GmbH, Rheinstetten, Germany) (700.13/176.04 MHz) spectrometers. HRESI and ESI mass spectra were recorded on an Agilent 6510 Q-TOF LC/MS mass spectrometer (Agilent Technologies, Santa Clara, CA, USA), and samples were dissolved in methanol (*c* 0.01 mg/mL). TLC was carried out on silica gel plates (CTX-1A, 5-17 µm, Sorbfil, Russia) and spots were visualized by spraying with aqueous 10% H_2_SO_4_ followed by heating. Column chromatography (CC) was performed on silica gel (KSK, 50−160 mesh, Sorbfil, Russia) and YMC ODS-A (12 nm, S-75 um, YMC Co., Ishikawa, Japan). HPLC was performed using an Agilent 1100 Series chromatograph with a differential refractometer (Agilent Technologies, Santa Clara, CA, USA). The reversed-phase columns (YMC-Pack ODS-A, YMC Co., Ishikawa, Japan, 10 mm × 250 mm, 5 µm and 4.6 mm × 250 mm, 5 µm) and normal-phase column (Ultrashere-Si, Beckman Instruments, Inc., Berkeley, CA, USA, 10 mm × 250 mm, 5 µm) were used for HPLC. Yields are based on dry weight of the sponge.

### 3.2. Animal Material

The samples of an *Inflatella* sp. sponge were collected by dredging near Kashevarov Bank, the Sea of Okhotsk (55°30′ N; 146°05′ E, Russia) at a depth of 214–197 m and were freeze dried after collection. The species was identified by Dr. Krasokhin V. B. from G.B. Elyakov Pacific Institute of Bioorganic Chemistry, FEB RAS. A voucher specimen (PIBOC O07-33(11)) is deposited at the G.B. Elyakov Pacific Institute of Bioorganic Chemistry, FEB RAS (Vladivostok, Russia).

### 3.3. Extraction and Isolation

The sponge material (dry weight 216 g) was chopped into pieces and extracted with EtOH at room temperature. The ethanol soluble materials (21.0 g) were obtained after concentration of the extract was dissolved in distilled water (300 mL) and partitioned in turn with CHCl_3_ (200 mL × 3). Evaporation of the chloroform extracts gave dark green gum (12.7 g) that was further separated on a silica gel column (7 cm × 10 cm) using the mixtures of CHCl_3_/EtOH as stepwise eluent systems to yield the fractions A–F (Figure S1).

Fraction D (301.4 mg), eluted with CHCl_3_/EtOH (15:1), was separated by column chromatography (CC) (YMC ODS-A, 15 mm × 100 mm, MeOH/H_2_O, 90–100%) to yield three subfractions (D1–D3). The subfraction D1 (128.5 mg) was further subjected to silica gel CC (hexane/EtOAc stepwise systems) to obtain three subfractions. The subfraction D1.2 (17.0 mg) was purified by normal-phase HPLC (Ultrasphere-Si, hexane/EtOAc, 1:1, 2 mL/min) to yield compound **13** (6.3 mg, 0.003%). Subfraction D1.3 (6.6 mg) was separated by reverse-phase HPLC (YMC-Pack ODS-A, 10 mm × 250 mm, 95% MeOH, 2 mL/min) to obtain compounds **1** (0.8 mg, 0.0004%), **3** (0.4 mg, 0.003%) and **7** (1.8 mg, 0.012%). 

Fraction E (904.4 mg), eluted with CHCl_3_/EtOH (10:1), was fractionated by silica gel CC (25 mm × 60 mm, hexane/EtOAc, 1:1) to yield four smaller fractions. Fraction E2 (227.4 mg) was subjected to normal-phase HPLC (Ultrasphere-Si, hexane/EtOAc, 1:1, 2 mL/min) resulting in three subfractions. The first subfraction E2.1 (4.0 mg) was purified using reverse-phase HPLC (YMC-Pack ODS-A, 4.6 mm × 250 mm, 90% EtOH, 0.6 mL/min) to afford compound **2** (0.8 mg, 0.0004%) and to re-isolate metabolite **13** (1.1 mg). As a result of the process of E2.2 (22.9 mg) reverse-phase separation (YMC-Pack ODS-A, 10 mm × 250 mm, 2 mL/min) in 95% MeOH, the compounds **4** (1.0 mg, 0.0005%), **6** (2.4 mg, 0.001%), **8** (6.3 mg, 0.003%), **10** (2.0 mg, 0.0009%) and **12** (1.7 mg, 0.0008%) were obtained. The last mentioned HPLC procedures and 94% MeOH as eluent were used for the subfraction E2.3 that allowed us to isolate the individual steroids **5** (6.8 mg, 0.003%), **9** (3.3 mg, 0.002%), **11** (8.0 mg, 0.004%), **14** (1.3 mg, 0.0006%) and to gain new portions of the metabolites **3** (5.8 mg) and **7** (24.3 mg).

#### 3.3.1. 24-Methylcholesta-6,24(28)-diene-3β,5α-diol (**1**)

White amorphous powder; [α]${}_{D}^{22}$ +45.0 (*c* 0.02, CHCl_3_); ^1^H and ^13^C NMR data (CDCl_3_), see Table 1 and Table 2; EIMS *m/z*: 414 [M]^+^ (10), 396 [M − H_2_O]^+^(100), 378 (15), 363 (20), 312 (8), 276 (8), 141 (12), 109 (14); (+) HRESI MS *m/z* 437.3393 [M + Na]^+^ (calcd. for C_28_H_46_O_2_Na, 437.3390).

#### 3.3.2. 24-Methylcholesta-5,24(28)-diene-3β,4α-diol (**2**)

White amorphous powder; [α]${}_{D}^{22}$ +50.0 (*c* 0.08, CHCl_3_); ^1^H and ^13^C NMR data (CDCl_3_), see Table 1 and Table 2; (+) HRESI MS *m/z* 437.3393 [M + Na]^+^ (calcd. for C_28_H_46_O_2_Na, 437.3390).

#### 3.3.3. (22*E*)-24-Nor-cholesta-5,22-diene-3β,7α-diol (**3**)

White amorphous powder; [α]${}_{D}^{22}$ −47.5 (*c* 0.04, CHCl_3_); ^1^H and ^13^C NMR data (CDCl_3_), see Table 1 and Table 2; (+) HRESI MS *m/z* 409.3081 [M+Na]^+^ (calcd. for C_26_H_42_O_2_Na, 409.3077).

#### 3.3.4. (22*E*)-24-Nor-cholesta-5,22-diene-3β,7β-diol (**4**)

White amorphous powder; [α]${}_{D}^{22}$ −9.0 (*c* 0.10, CHCl_3_); ^1^H and ^13^C NMR data (CDCl_3_), see Table 1 and Table 2; (+) HRESI MS *m/z* 409.3081 [M + Na]^+^ (calcd. for C_26_H_42_O_2_Na, 409.3077).

### 3.4. Biological Activities

#### 3.4.1. Cell Line and Culture Condition

The neuroblastoma cell line Neuro2a was purchased from ATCC. Cells were cultured according to the manufacturer instructions in DMEM medium containing 10% fetal bovine serum (Biolot, Russia) and 1% penicillin/streptomycin (Invitrogen). Cells were incubated at 37 °C in a humidified atmosphere containing 5% (*v*/*v*) CO_2_.

#### 3.4.2. MTT Assay

Cell suspension (1 × 10^3^ cells/well) was incubated with different concentration of compounds during 24 h. After that, cell viability was determined by MTT (3-(4,5-dimethylthiazol-2-yl)-2,5- diphenyltetrazolium bromide) method as manufacturer described (Sigma-Aldrich, St. Louis, MO, USA). 

#### 3.4.3. Nonspecific Esterase Activity Assay

Cell suspension (1 × 10^3^ cells/well) was incubated with different concentrations of the studied compounds during 24 h. A stock solution of the fluorescein diacetate (FDA) (Sigma-Aldrich, USA) in DMSO (1 mg/mL) was prepared. After incubation of the cells with compounds, FDA solution (50 μg/mL) was added to each well and the plate was incubated at 37 °C for 15 min. Cells were washed with phosphate buffer saline and fluorescence was measured with a Fluoroskan Ascent plate reader (ThermoLabsystems, Finland) at λ_ex_ = 485 nm and λ_em_ = 518 nm. Cell viability was expressed as the percent of control.

#### 3.4.4. 6-OHDA-Induced In Vitro Model of Parkinson’s Disease

The neuroprotective activity of the studied compounds in 6-hydroxydopamine-induced cell model of Parkinson’s disease was investigated as described previously [37]. Neuroblastoma Neuro2a line cells (1 × 10^3^ cells/well) were treated with compounds at concentrations of 1 and 10 µM during 1 h, after that 6-OHDA (Sigma-Aldrich, USA) at concentration of 50 µM was added in each well and neuroblastoma cells were cultivated during 24 h. After that, viability of cells was measured by MTT assay. The results were presented as percent of control data.

#### 3.4.5. Reactive Oxygen Species (ROS) Level Analysis

Cell suspensions (1 × 10^3^ cells/well) were incubated during 1 h with 1 and 10 µM solutions of the tested compounds. Non-treated and treated with 6-OHDA at concentration of 50 µM (Sigma-Aldrich, USA) cells were used as negative and positive controls, respectively. The portion (20 µL) of 2′,7′-dichlorodihydrofluorescein diacetate (H2DCF-DA) stock solution (Molecular Probes, Eugene, OR, USA) with concentration of 100 mM was added in each well and the microplate was incubated for an additional 10 min at 37 °C. The intensity of dichlorofluorescin fluorescence was measured at λ_ex_ = 485 nm, and λ_em_ = 518 nm with plate reader PHERAstar FS (BMG Labtech, Ortenberg, Germany). The data were processed by MARS Data Analysis v. 3.01R2 (BMG Labtech, Ortenberg, Germany). In other experiments, cells were incubated with compounds during 1 h. Then, 6-OHDA (50 µM) was added in each well for 30 min and ROS levels were measured. All obtained results were presented as percent of negative control data.

## 4. Conclusions

A sponge *Inflatella* sp. contains a variety of oxysterols differing from each other in positions of additional hydroxy or oxo groups of their tetracyclic core and in structures of side chains. Fourteen oxidized sterols, including four previously unknown compounds, were isolated. Structures of new oxysterols have been established. Previously known compounds were structurally identified by comparison of their NMR and MS spectra with those reported in literature. All of the obtained compounds were studied in the 6-hydroxydopamine-induced cell model of Parkinson’s disease. At least, one new oxysterol, (22*E*)-24-*nor*-cholesta-5,22-diene-3β,7β-diol (**4**), showed a substantial activity in this test and might be used for the further studies as a drug candidate.

## Figures and Tables

**Table 1:** ^13^C NMR data of Compounds **1–4** (δ in ppm, CDCl_3_).

No.	1 ^a^	2 ^a^	3 ^b^	4 ^a^
1	28.5	36.7	37.0	37.0
2	30.8	28.1	31.4	31.6
3	67.4	76.6	71.4	71.5
4	41.2	75.2	42.1	41.8
5	74.2	142.1	146.2	143.7
6	133.5	117.8	123.9	125.5
7	133.4	31.6	65.3	73.4
8	38.7	31.5	37.5	40.9
9	45.3	50.5	42.3	48.3
10	38.3	38.1	37.4	36.5
11	21.3	20.9	20.7	21.1
12	40.3	39.8	39.1	39.5
13	44.0	42.3	42.0	42.8
14	54.2	56.8	49.5	56.0
15	24.1	24.3	24.2	26.3
16	28.8	28.2	28.4	28.6
17	56.1	56.0	55.8	55.4
18	12.4	11.9	11.9	12.1
19	14.9	20.2	18.2	19.2
20	36.0	35.8	39.8	39.7
21	18.9	18.7	20.8	20.9
22	34.9	34.7	133.6	133.5
23	31.3	31.0	134.9	135.0
24	157.1	156.9		
25	34.1	33.8	30.9	30.9
26	22.1	21.9	22.7	22.7
27	22.2	22.0	22.7	22.7
28	106.3	106.0		

^a^ Spectra recorded at 125.76 MHz; ^b^ spectra recorded at 176.04 MHz.

**Table 2:** ^1^H NMR data of Compounds **1–4** (δ in ppm, *J* in Hz, CDCl_3_).

No. ^a^	1 ^b^	2 ^c^	3 ^c^	4 ^b^
1	α: 1.62, m β: 1.37, m	α: 1.14, m β: 1.84, m	α: 1.12, m β: 1.87, m	α: 1.06, m β: 1.85, m
2	α: 1.89, m β: 1.55, m	α: 1.89, m β: 1.60, m	α: 1.86, m β: 1.52, m	α: 1.85, m β: 1.52, m
3	4.12, m	3.27, td (10.5, 4.9)	3.39, m	3.55, m
4	α: 1.80, dd (12.4, 4.9) β: 1.64, m	4.06, m	α: 2.34, ddd (13.2, 4.9, 2.2) β: 2.29, ddt (13.2, 11.3, 1.8)	α: 2.34, ddd (13.2, 5.2, 2.3) β: 2.26, ddt (13.2, 11.3, 2.2)
5				
6	5.57, dd (9.8, 2.7)	5.74, dt (5.5, 2.2)	5.60, dd (5.3, 1.6)	5.29, br t (2.2)
7	5.63, dd (9.8, 1.6)	α: 1.58, m β: 2.10, m	3.85, br s	3.84, dt (8.3, 2.2)
8	1.93, m	1.44, m	1.47, m	1.40, m
9	1.48, dd (11.6, 3.9)	0.99, m	1.23, m	1.03, m
10				
11	α: 1.44, m β: 1.31, m	α: 1.01, m β: 1.48, m	α: 1.54, m β: 1.49, m	α: 1.53, m β: 1.47, m
12	α: 1.23, m β: 2.05, dt (12.5, 3.1)	α: 1.18, td (12.7, 5.2) β: 2.03, dt (12.7, 3.7)	α: 1.19, m β: 1.98, dt (12.6, 3.5)	α: 1.15, m β: 1.99, m
13				
14	1.23, m	1.00, m	1.44, m	1.16, m
15	α: 1.67, m β: 1.22, m	α: 1.61, m β: 1.10, m	α: 1.67, m β: 1.12, m	α: 1.77, m β: 1.40, m
16	α: 1.89, m β: 1.32, m	α: 1.87, m β: 1.29, m	α: 1.73, m β: 1.29, m	α: 1.71, m β: 1.30, m
17	1.17, m	1.14, m	1.20, m	1.12, m
18	0.71, s	0.69, s	0.70, s	0.70, s
19	0.92, s	1.03, s	1.00, s	1.35, s
20	1.43, m	1.42, m	2.01, m	2.00, m
21	0.96, d (6.5)	0.95, d (6.6)	1.01, d (6.7)	1.01, d (6.6)
22	1.16, m 1.55, m	1.16, m 1.55, m	5.18, dd (15.4, 8.4)	5.18, dd (15.4, 8.4)
23	1.88, m 2.10, ddd (15.4, 11.5, 4.8)	1.88, m 2.09, m	5.27, dd (15.3, 6.6)	5.28, dd (15.4, 6.5)
24				
25	2.23, sept (6.8)	2.23, sept (7.0)	2.19, br sept (6.7)	2.19, br sept (6.7)
26	1.03, d (6.8)	1.02, d (7.0)	0.94, d (6.7)	0.95, d (6.7)
27	1.02, d (6.8)	1.03, d (7.0)	0.94, d (6.7)	0.95, d (6.7)
28	4.66, s 4.72, s	4.66, s 4.72, s		

^a^ Assignments were made with the aid of the ^1^H-^1^H COSY and HSQC spectra; ^b^ spectra recorded at 500.13 MHz; ^c^ spectra recorded at 700.13 MHz.

## Supplementary Materials

^1^H and ^13^C spectra of new compounds **1**–**4** are available online at http://www.mdpi.com/1660-3397/16/11/458/s1, Figure S1: Experimental Section, Figure S2: HRESI MS Spectra (Positive Ion Mode) of compounds **1**–**4** in CDCl_3_, Figure S3: ^1^H NMR (500.13 MHz) spectrum of **1** in CDCl_3_, Figure S4: ^13^C NMR (125.76 MHz) spectrum of **1** in CDCl_3_, Figure S5: COSY NMR (700.13 MHz) spectrum of **1** in CDCl_3_, Figure S6: ROESY NMR (500.13 MHz) spectrum of **1** in CDCl_3_, Figure S7: HSQC NMR (700.13 MHz) spectrum of **1** in CDCl_3_, Figure S8: ^1^H NMR (700.13 MHz) spectrum of **2** in CDCl_3_, Figure S9: ^13^C NMR (125.76 MHz) spectrum of **2** in CDCl_3_, Figure S10: COSY NMR (700.13 MHz) spectrum of **2** in CDCl_3_, Figure S11: ROESY NMR (700.13 MHz) spectrum of **2** in CDCl_3_, Figure S12: HSQC NMR (700.13 MHz) spectrum of **2** in CDCl_3_, Figure S13: ^1^H NMR (700.13 MHz) spectrum of **3** in CDCl_3_, Figure S14: ^13^C NMR (176.04 MHz) spectrum of **3** in CDCl_3_, Figure S15: COSY NMR (700.13 MHz) spectrum of **3** in CDCl_3_, Figure S16: ROESY NMR (700.13 MHz) spectrum of **3** in CDCl_3_, Figure S17: HSQC NMR (700.13 MHz) spectrum of **3** in CDCl_3_, Figure S18: HMBC NMR (700.13 MHz) spectrum of **3** in CDCl_3_, Figure S19: ^1^H NMR (500.13 MHz) spectrum of **4** in CDCl_3_, Figure S20: ^13^C NMR (125.76 MHz) spectrum of **4** in CDCl_3_, Figure S21: COSY NMR (500.13 MHz) spectrum of **4** in CDCl_3_, Figure S22: ROESY NMR (500.13 MHz) spectrum of **4** in CDCl_3_, Figure S23: HSQC NMR (500.13 MHz) spectrum of **4** in CDCl_3_, S24: HSQC NMR (500.13 MHz) spectrum of **4** in CDCl_3_, Figure S25: Viability of Neuro2a cells, Figure S26 ROS formation in Neuro2a cells, Figure S27: Viability of Neuro2a cells treated with 6-OHDA.
