# Supplementary material for: Oxysterols from a Marine Sponge Inflatella sp. and Their Action in 6-Hydroxydopamine-Induced Cell Model of Parkinson’s Disease
Source: Mar Drugs. 2018 Nov 21;16(11):458. doi: 10.3390/md16110458 (PMC6267020; doi:10.3390/md16110458)
Supplement: Supplementary file 1 [file marinedrugs-16-00458-s001.pdf]

Supplementary data contents page

Title: Oxysterols from a Marine Sponge *Inflatella* sp. and their Action in 6-Hydroxydopamine-Induced Cell Model of Parkinson's Disease

Authors: Sophia A. Kolesnikova[1,*,†], Ekaterina G. Lyakhova[1,†], Anatoly I. Kalinovsky[1], Roman S. Popov[1], Ekaterina A. Yurchenko[1], and Valentin A. Stonik[1,2]

Address: [1]*G.B. Elyakov Pacific Institute of Bioorganic Chemistry, Far Eastern Branch of Russian Academy of Sciences, Pr. 100-let Vladivostoku 159, 690022 Vladivostok, Russia*

[2]*School of Natural Science, Far Eastern Federal University, Sukhanova St., 8, Vladivostok 690000, Russia*

**Correspondence: ***sovin81@inbox.ru; Tel.: +7-423-231-1168

Contents:

S1 Experimental Section
S2 HRESI MS Spectra (Positive Ion Mode) of compounds **1**-**4** in $CDCl_3$
S3 ^{1}H NMR (500.13 MHz) spectrum of the 24-methylcholesta-6,24(28)-diene-3β,5α-diol (**1**) in $CDCl_3$
S4 ^{13}C NMR (125.76 MHz) spectrum of the 24-methylcholesta-6,24(28)-diene-3β,5α-diol (**1**) in $CDCl_3$
S5 COSY NMR (700.13 MHz) spectrum of the 24-methylcholesta-6,24(28)-diene-3β,5α-diol (**1**) in $CDCl_3$
S6 ROESY NMR (500.13 MHz) spectrum of the 24-methylcholesta-6,24(28)-diene-3β,5α-diol (**1**) in $CDCl_3$
S7 HSQC NMR (700.13 MHz) spectrum of the 24-methylcholesta-6,24(28)-diene-3β,5α-diol (**1**) in $CDCl_3$
S8 ^{1}H NMR (700.13 MHz) spectrum of the 24-methylcholesta-5,24(28)-diene-3β,4α-diol (**2**) in $CDCl_3$
S9 ^{13}C NMR (125.76 MHz) spectrum of the 24-methylcholesta-5,24(28)-diene-3β,4α-diol (**2**) in $CDCl_3$
S10 COSY NMR (700.13 MHz) spectrum of the 24-methylcholesta-5,24(28)-diene-3β,4α-diol (**2**) in $CDCl_3$
S11 ROESY NMR (700.13 MHz) spectrum of the 24-methylcholesta-5,24(28)-diene-3β,4α-diol (**2**) in $CDCl_3$
S12 HSQC NMR (700.13 MHz) spectrum of the 24-methylcholesta-5,24(28)-diene-3β,4α-diol (**2**) in $CDCl_3$
S13 ^{1}H NMR (700.13 MHz) spectrum of the (22*E*)-24-*nor*-cholesta-5,22-diene-3β,7α-diol (**3**) in $CDCl_3$
S14 ^{13}C NMR (176.04 MHz) spectrum of the (22*E*)-24-*nor*-cholesta-5,22-diene-3β,7α-diol (**3**) in $CDCl_3$
S15 COSY NMR (700.13 MHz) spectrum of the (22*E*)-24-*nor*-cholesta-5,22-diene-3β,7α-diol (**3**) in $CDCl_3$
S16 ROESY NMR (700.13 MHz) spectrum of the (22*E*)-24-*nor*-cholesta-5,22-diene-3β,7α-diol (**3**) in $CDCl_3$
S17 HSQC NMR (700.13 MHz) spectrum of the (22*E*)-24-*nor*-cholesta-5,22-diene-3β,7α-diol (**3**) in $CDCl_3$
S18 HMBC NMR (700.13 MHz) spectrum of the (22*E*)-24-*nor*-cholesta-5,22-diene-3β,7α-diol (**3**) in $CDCl_3$
S19 ^{1}H NMR (500.13 MHz) spectrum of the (22*E*)-24-*nor*-cholesta-5,22-diene-3β,7β-diol (**4**) in $CDCl_3$
S20 ^{13}C NMR (125.76 MHz) spectrum of the (22*E*)-24-*nor*-cholesta-5,22-diene-3β,7β-diol (**4**) in $CDCl_3$
S21 COSY NMR (500.13 MHz) spectrum of the (22*E*)-24-*nor*-cholesta-5,22-diene-3β,7β-diol (**4**) in $CDCl_3$
S22 ROESY NMR (500.13 MHz) spectrum of the (22*E*)-24-*nor*-cholesta-5,22-diene-3β,7β-diol (**4**) in $CDCl_3$
S23 HSQC NMR (500.13 MHz) spectrum of the (22*E*)-24-*nor*-cholesta-5,22-diene-3β,7β-diol (**4**) in $CDCl_3$
S24 HMBC NMR (500.13 MHz) spectrum of the (22*E*)-24-*nor*-cholesta-5,22-diene-3β,7β-diol (**4**) in $CDCl_3$
S25 Viability of Neuro2a cells
S26 ROS formation in Neuro2a cells
S27 Viability of Neuro2a cells treated with 6-OHDA

† These authors contributed equally to the work.

S1 Experimental Section

General experimental procedure:

^{1}H NMR (500.13, 700.13 MHz) and ^{13}C NMR (125.75 MHz, 176.04 MHz) spectra were recorded in $CDCl_3$ on a Avance-III 700 and DRX-500 «Bruker». The ^{1}H and ^{13}C NMR chemical shifts were referenced to the TMS or to solvent peak for $CDCl_3$ at δ_H 7.26 and δ_C 77.0.

HRESI-MS: Agilent 6510 Q-TOF LC/MS, 0.01 mg/ml ($CDCl_3$), 5µl min^{-1}. LSI-MS: AMD-604 (AMD Intectra), 1 mg/ml. HPLC: YMC-Pack ODS-A (5μ, 250×4.6 mm).

TLC examination of fractions A-F :

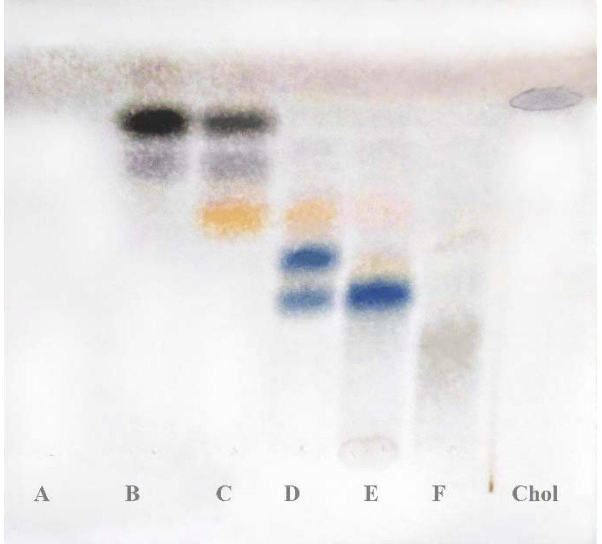

TLC was carried out on silica gel plates (CTX-1A, 5-17 µm, Sorbfil, Russia), $CHCl_3$/EtOH (25:1).

Statistical analysis

All assays were performed at least in triplicate. The results are expressed as the mean ± standard deviation (SD). A Student's *t*-test was used to evaluate the data with the significance level of $p < 0.05$. The means and standard errors for each treatment were calculated and plotted using SigmaPlot 3.02 software (Jandel Scientific, San Rafael, CA).

S2 HRESI MS Spectra (Positive Ion Mode) of compounds **1-4** in $CDCl_3$

1

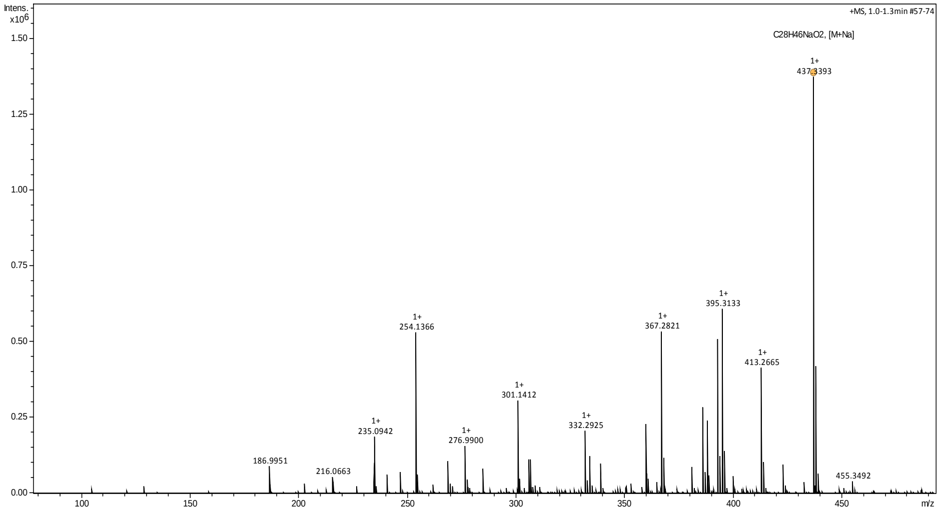

2

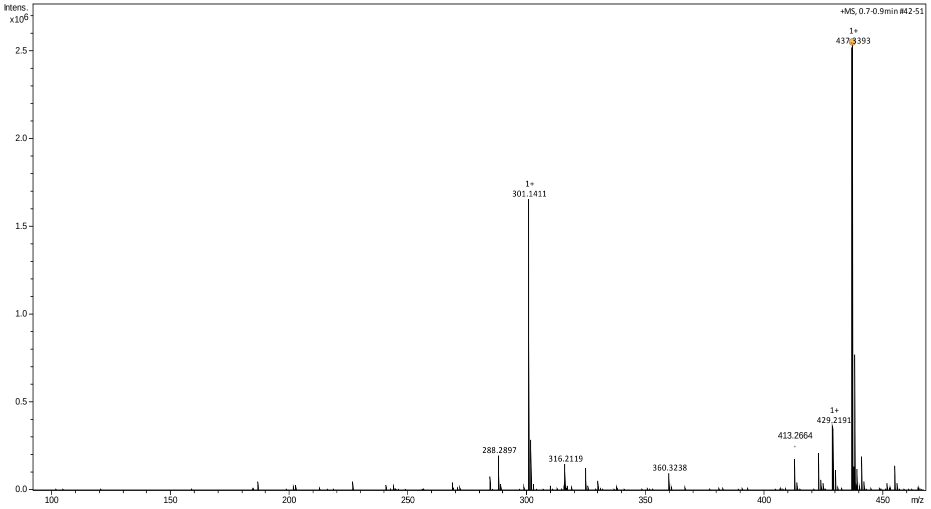

3

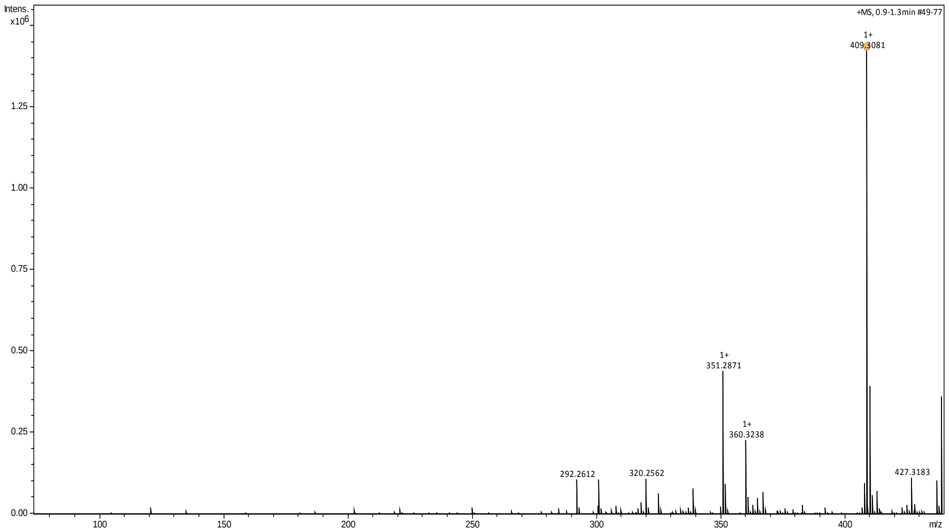

4

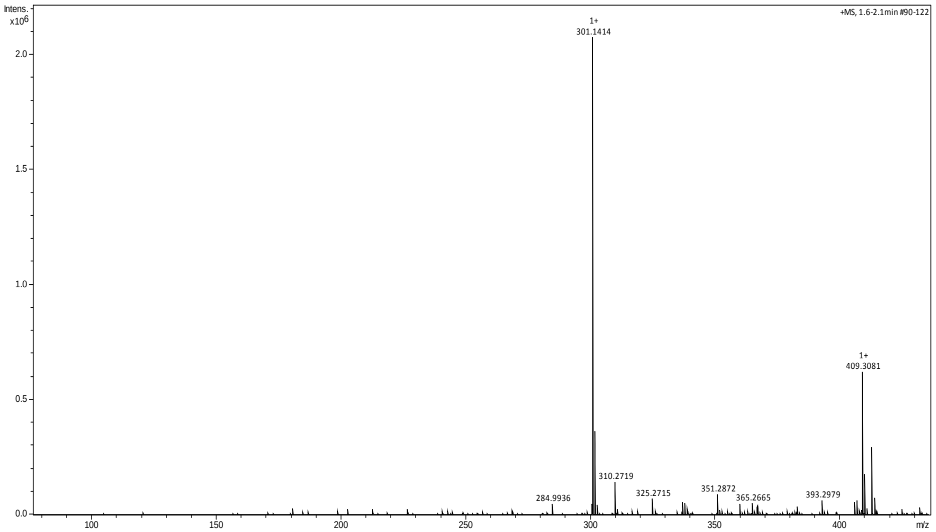

S3 ^{1}H NMR (700.13 MHz) spectrum for the 24-methylcholesta-6,24(28)-diene-3β,5α-diol (**1**) in $CDCl_3$

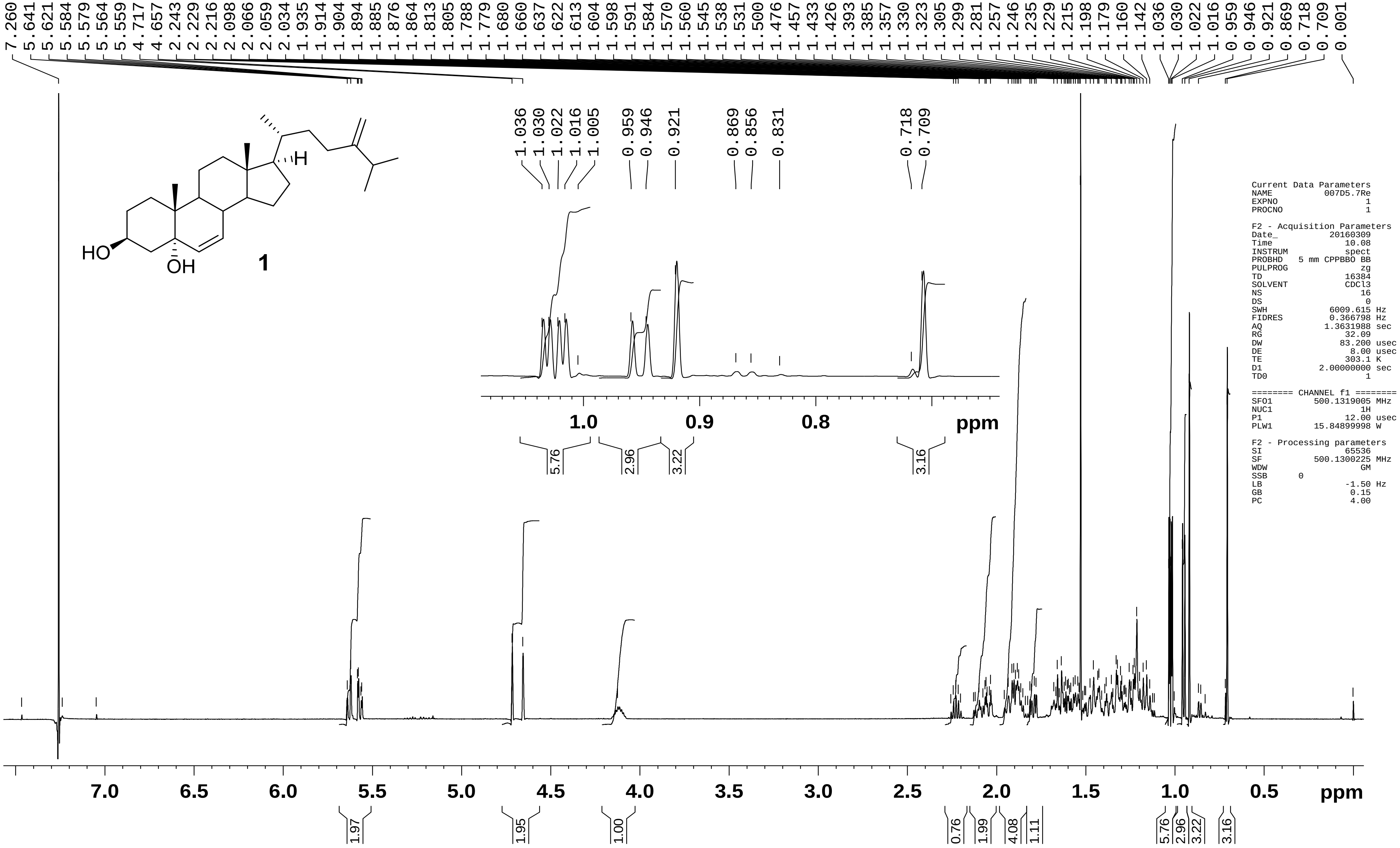

S4 ^{13}C NMR (176.04 MHz) spectrum for the 24-methylcholesta-6,24(28)-diene-3β,5α-diol (**1**) in $CDCl_3$

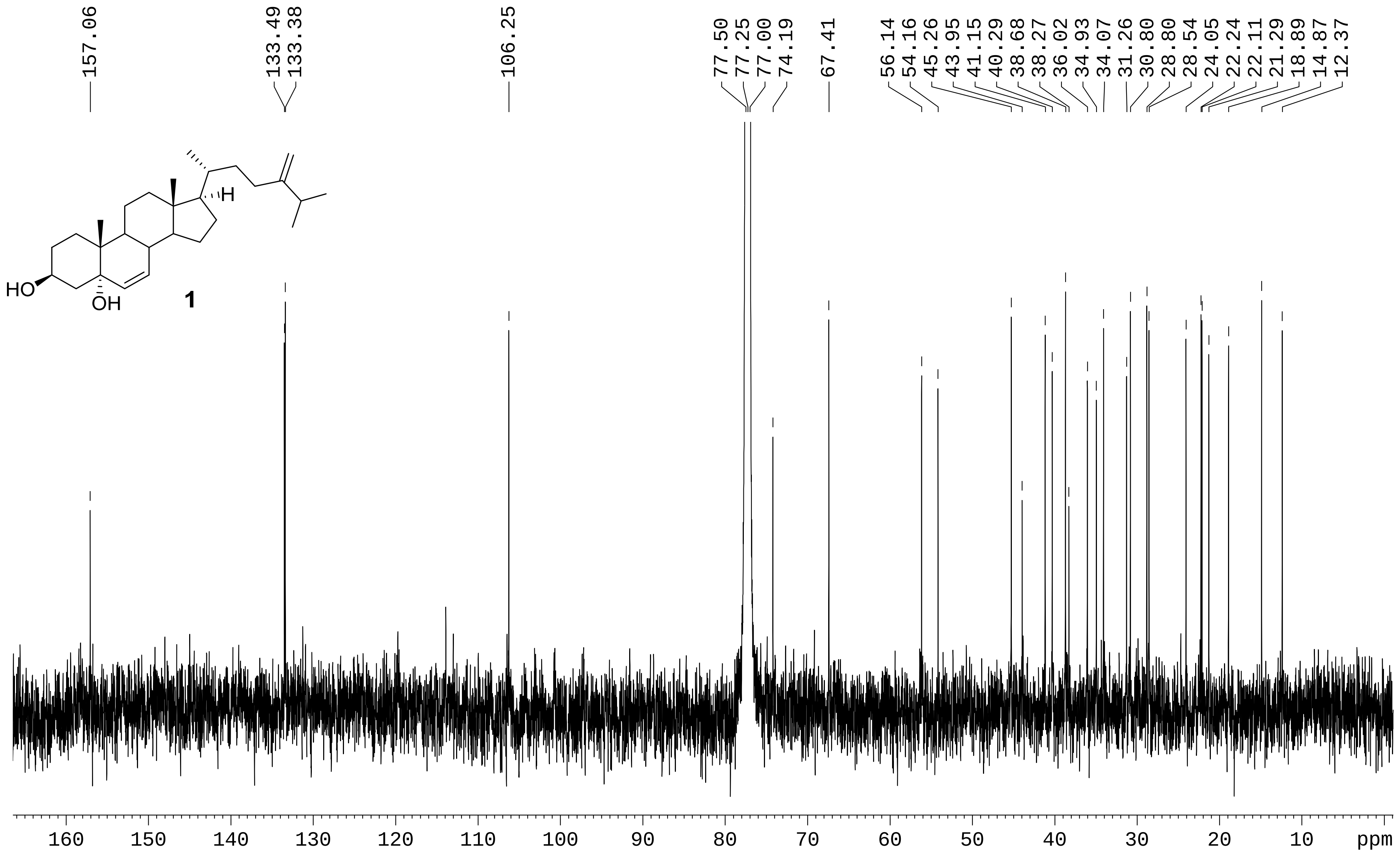

S5 COSY NMR (700.13 MHz) spectrum of the 24-methylcholesta-6,24(28)-diene-3β,5α-diol (**1**) in $CDCl_3$

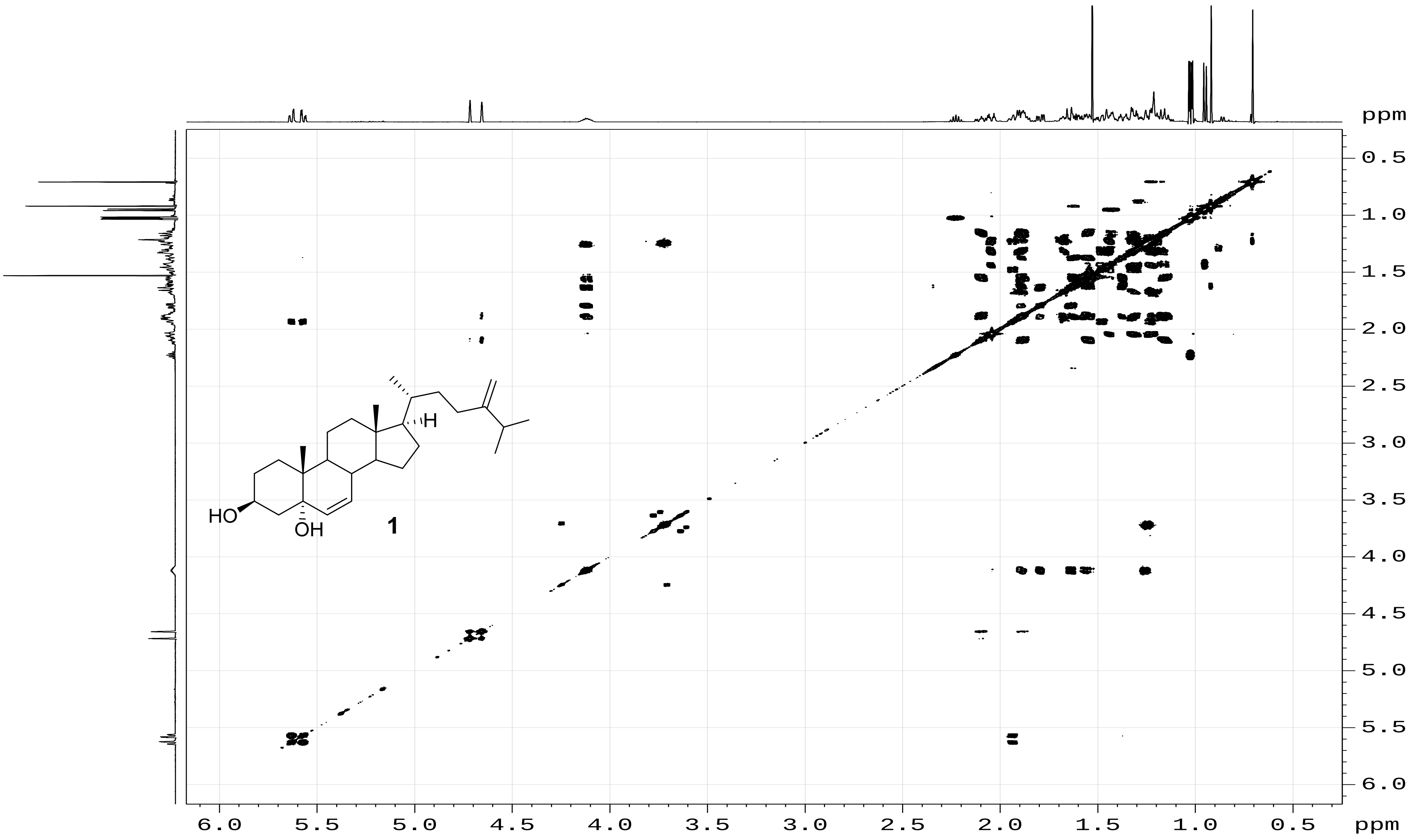

S6 ROESY NMR (500.13 MHz) spectrum of the 24-methylcholesta-6,24(28)-diene-3β,5α-diol (**1**) in $CDCl_3$

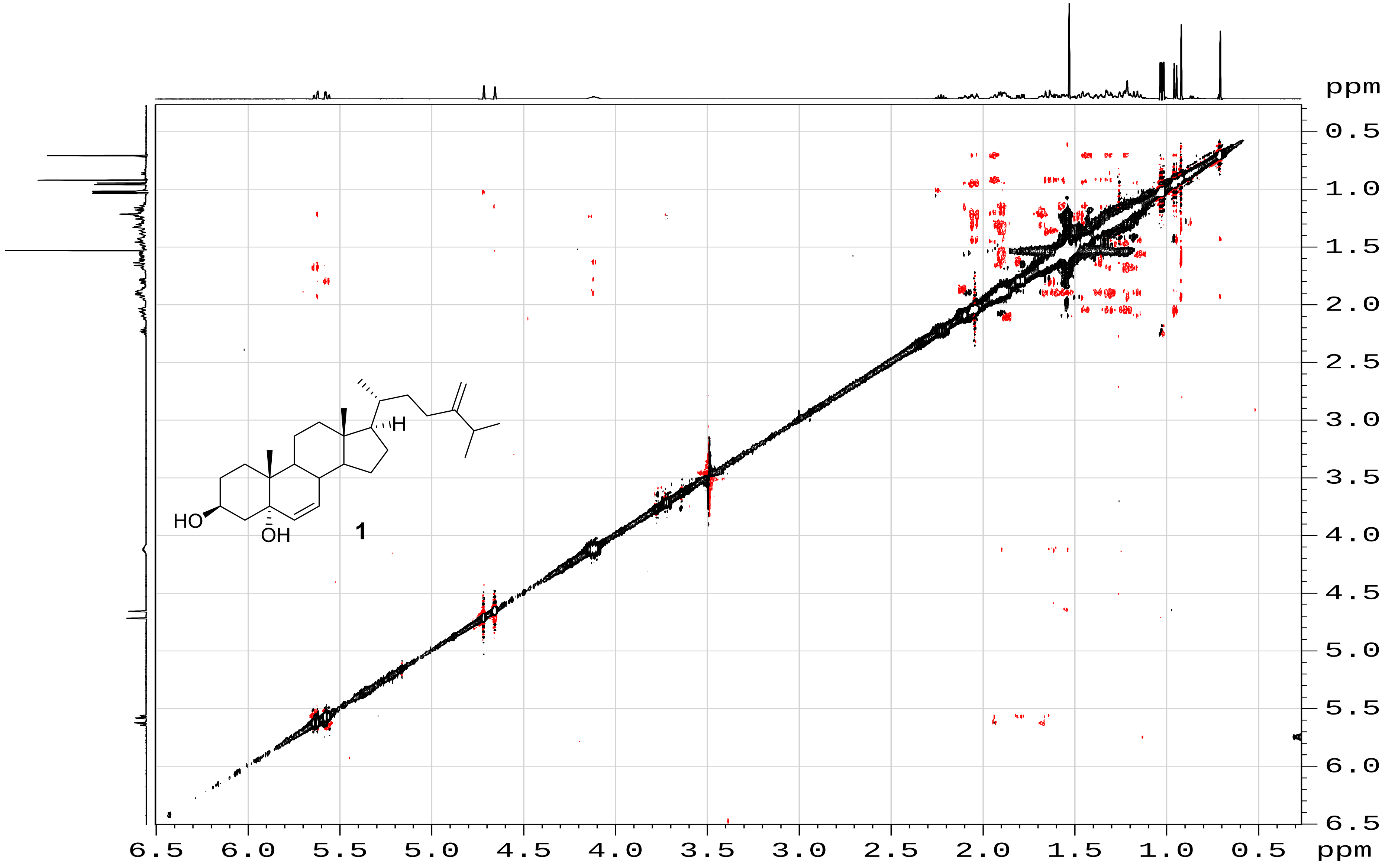

S7 HSQC NMR (700.13 MHz) spectrum of the 24-methylcholesta-6,24(28)-diene-3β,5α-diol (**1**) in $CDCl_3$

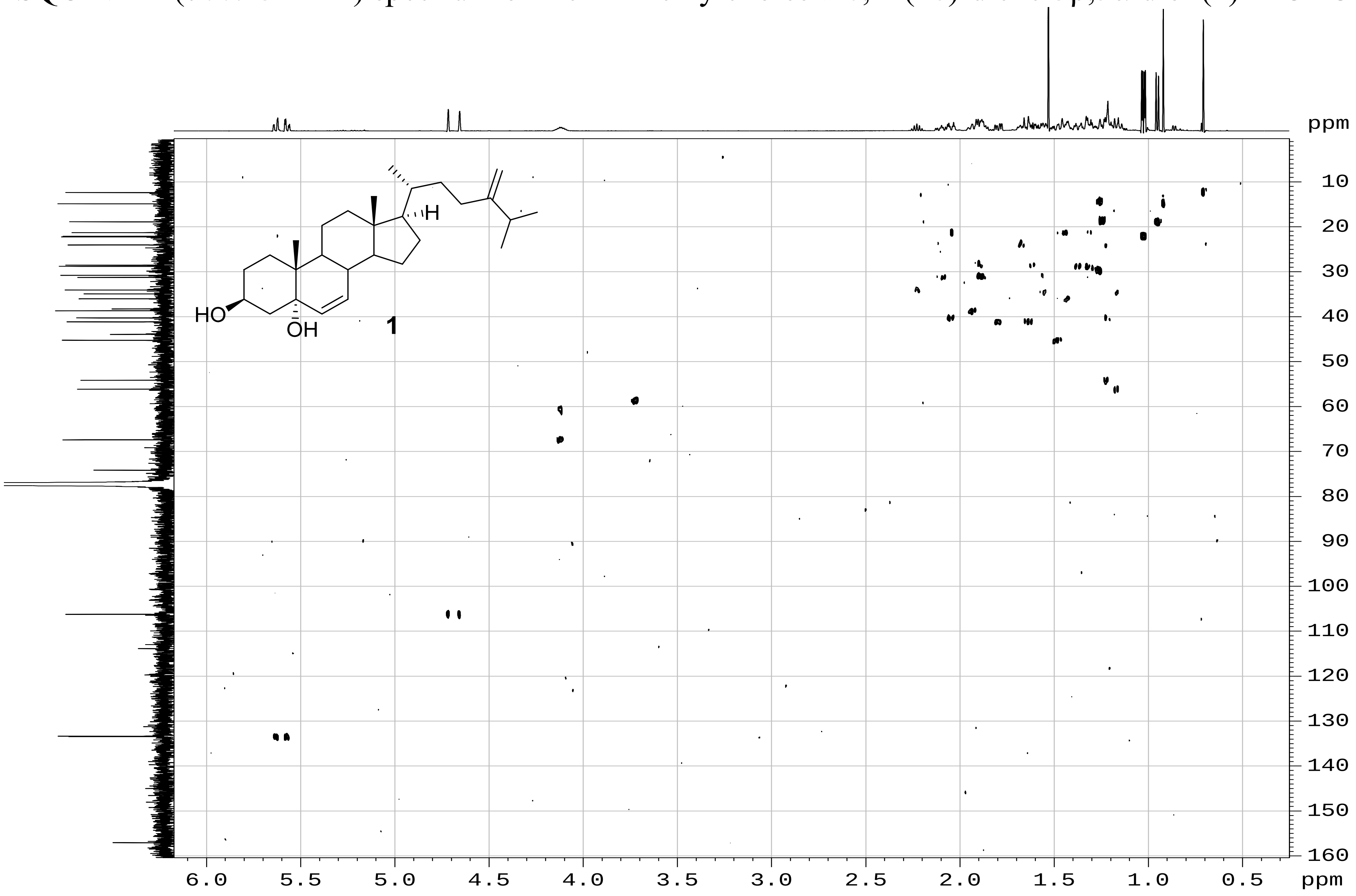

S 8 ^{1}H NMR (700.13 MHz) spectrum of the 24-methylcholesta-5,24(28)-diene-3β,4α-diol (**2**) in $CDCl_3$

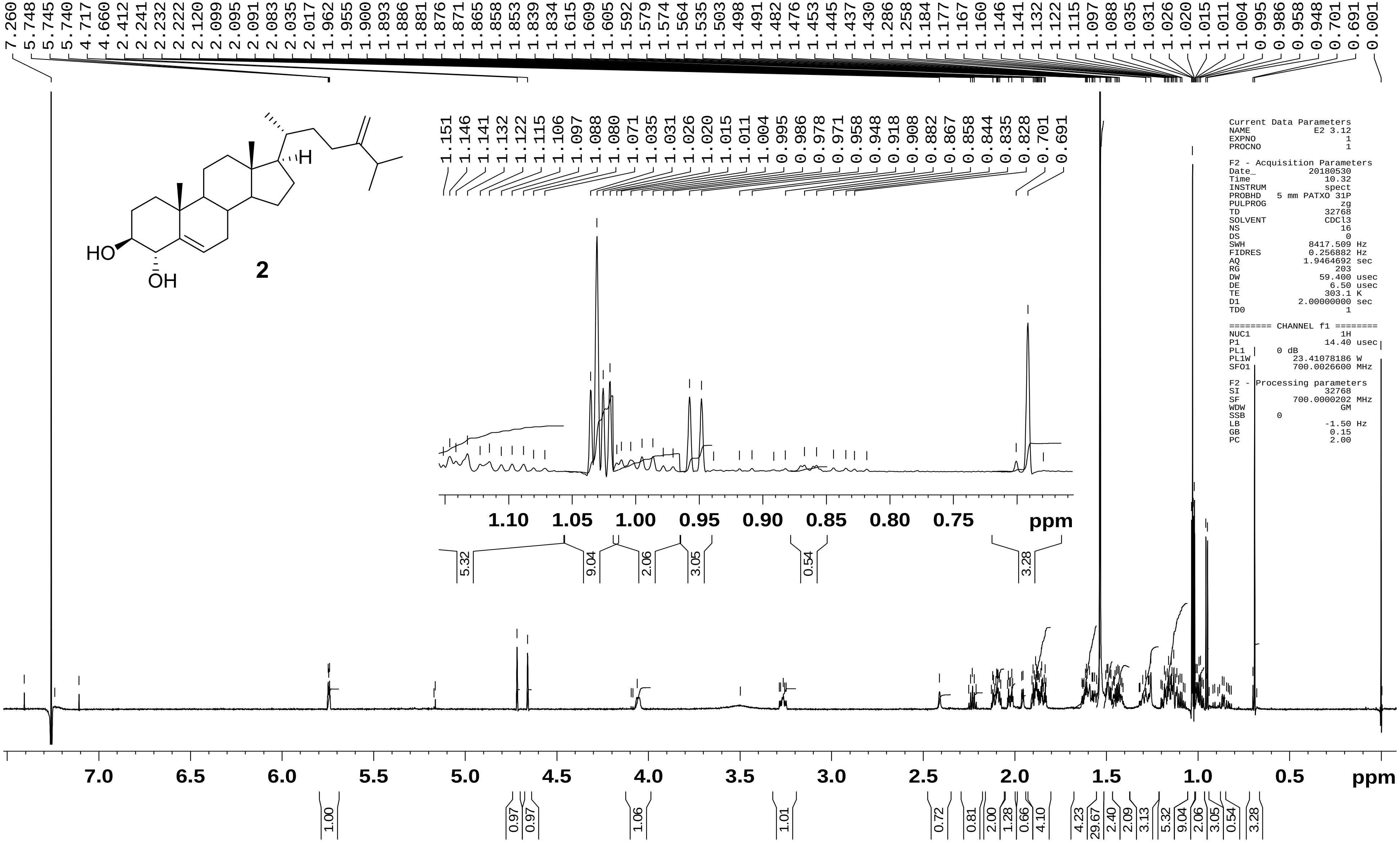

S9 ^{13}C NMR (176.04 MHz) spectrum of the 24-methylcholesta-5,24(28)-diene-3β,4α-diol (**2**) in $CDCl_3$

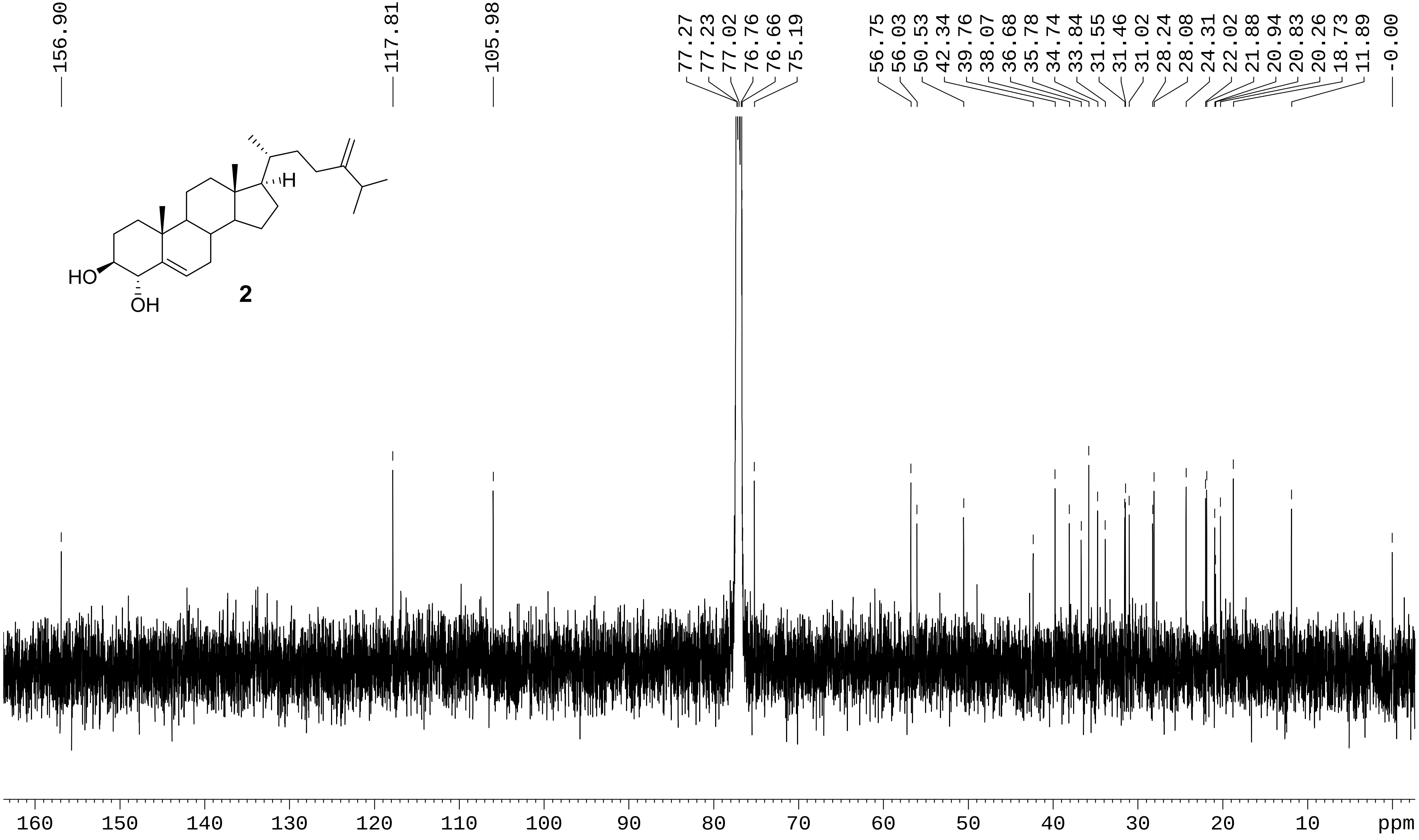

S10 COSY NMR (700.13 MHz) spectrum of the 24-methylcholesta-5,24(28)-diene-3β,4α-diol (**2**) in $CDCl_3$

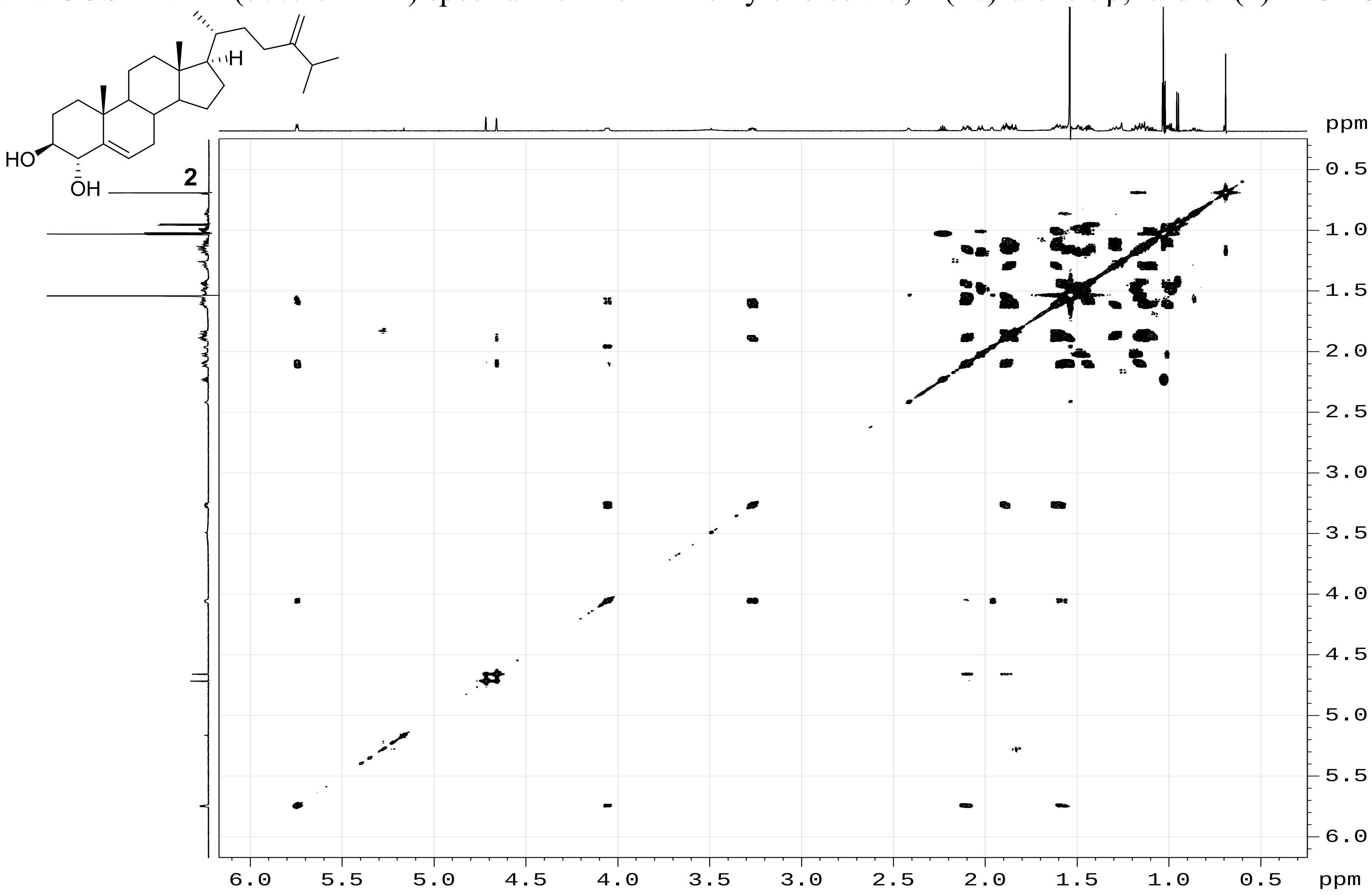

S11 ROESY NMR (500.13 MHz) spectrum of the 24-methylcholesta-5,24(28)-diene-3β,4α-diol (**2**) in $CDCl_3$

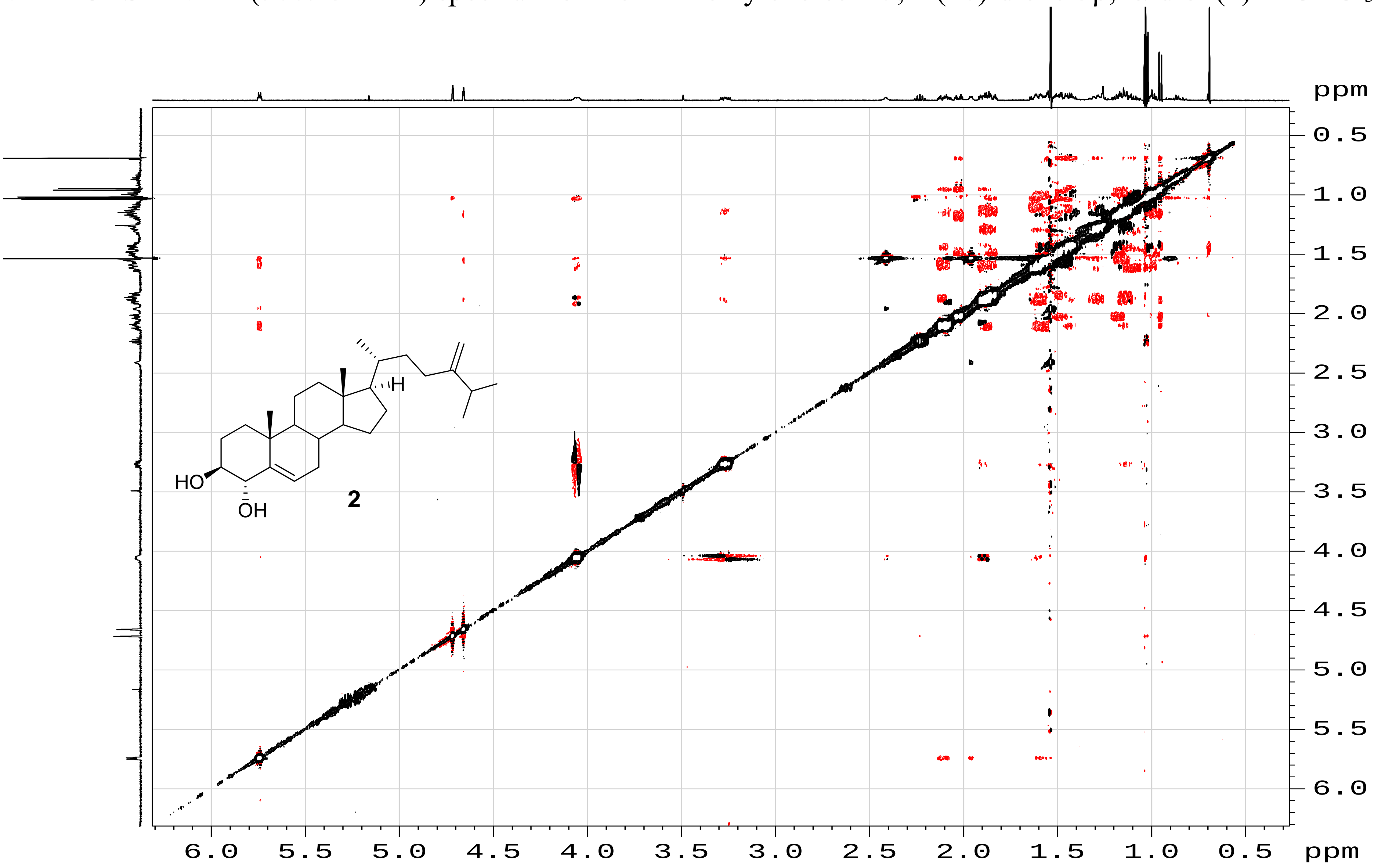

S12 HSQC NMR (700.13 MHz) spectrum for the 24-methylcholesta-5,24(28)-diene-3β,4α-diol (**2**) in $CDCl_3$

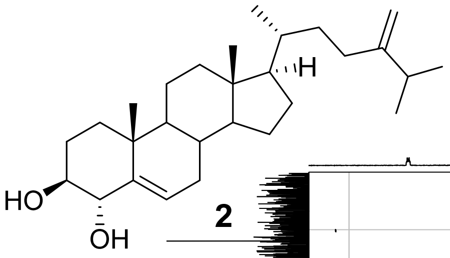

2

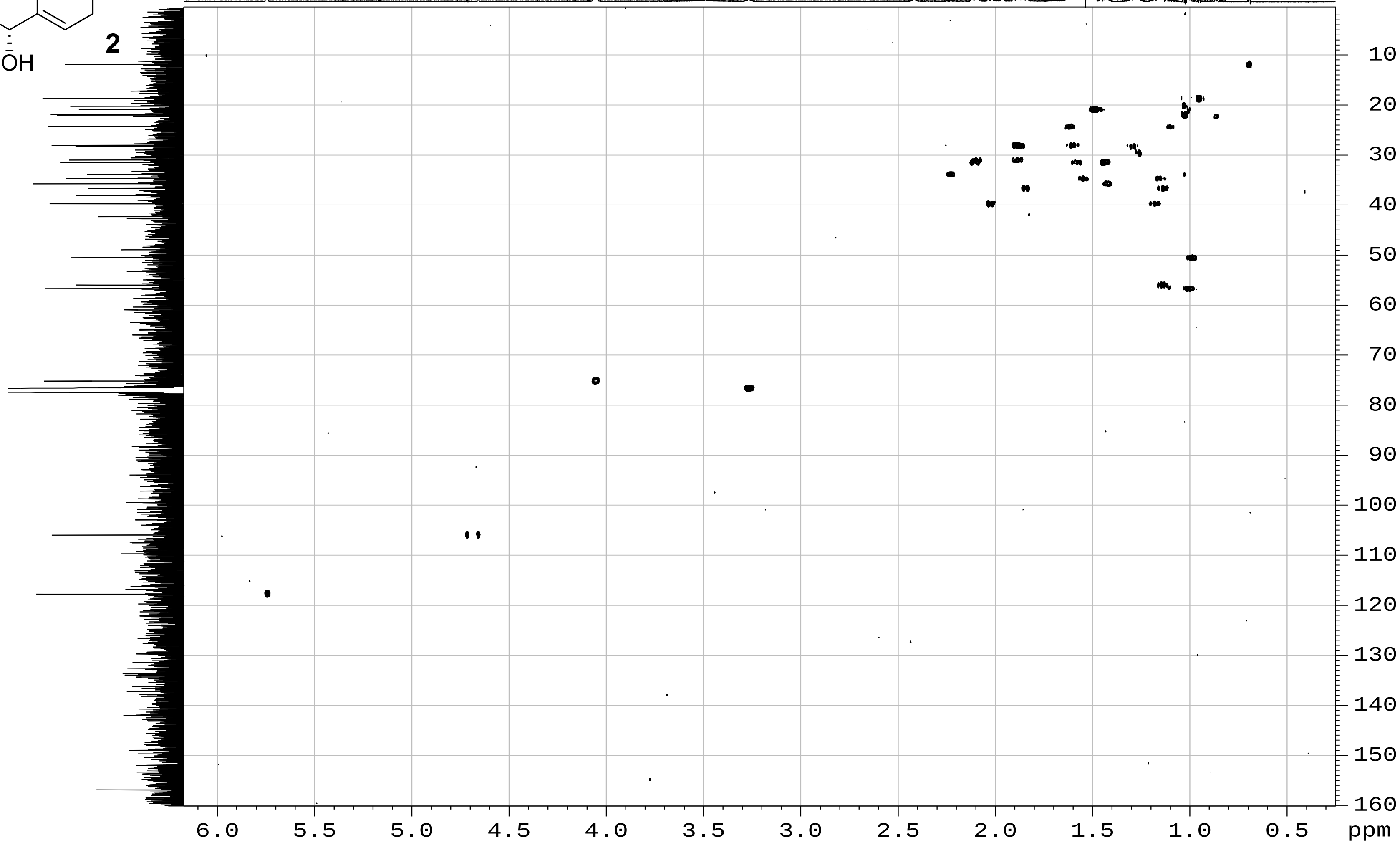

S13 ^{1}H NMR (700.13 MHz) spectrum of the (22*E*)-24-*nor*-cholesta-5,22-diene-3β,7α-diol (**3**) in $CDCl_3$

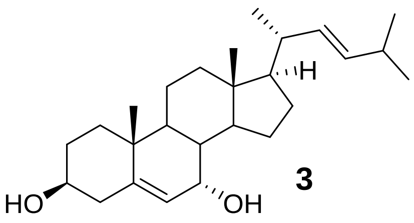

3

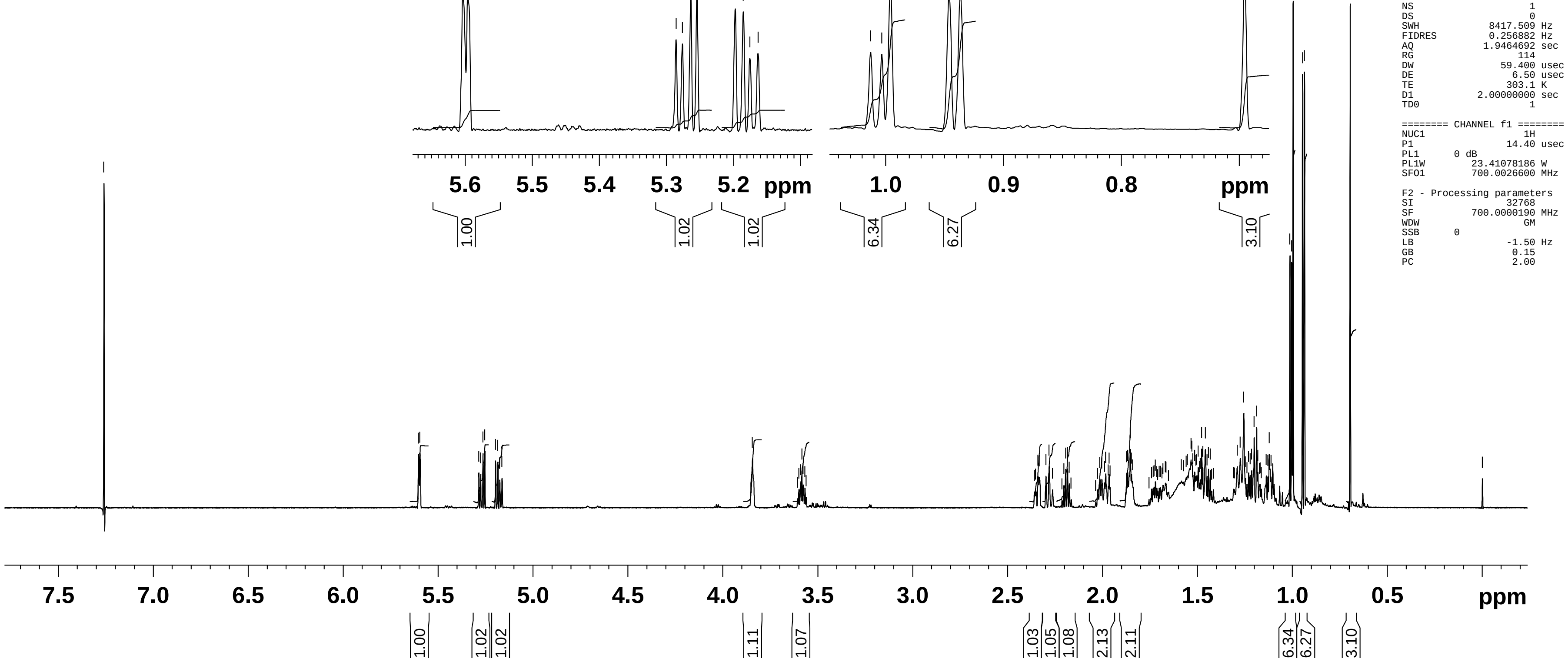

S14 ^{13}C NMR (176.04 MHz) spectrum of the (22*E*)-24-*nor*-cholesta-5,22-diene-3β,7α-diol (**3**) in $CDCl_3$

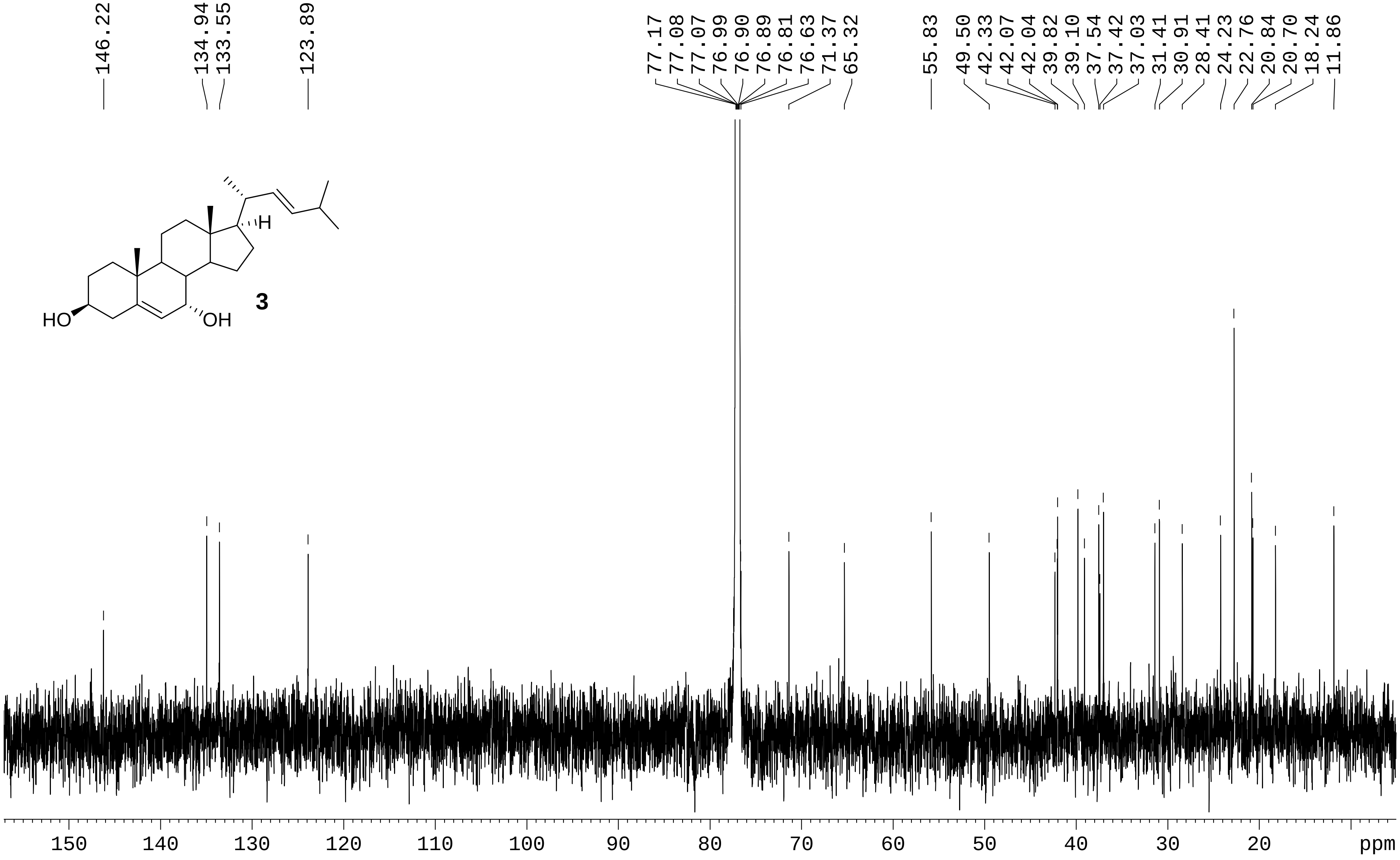

S15 COSY NMR (700.13 MHz) spectrum of the (22*E*)-24-*nor*-cholesta-5,22-diene-3β,7α-diol (**3**) in $CDCl_3$

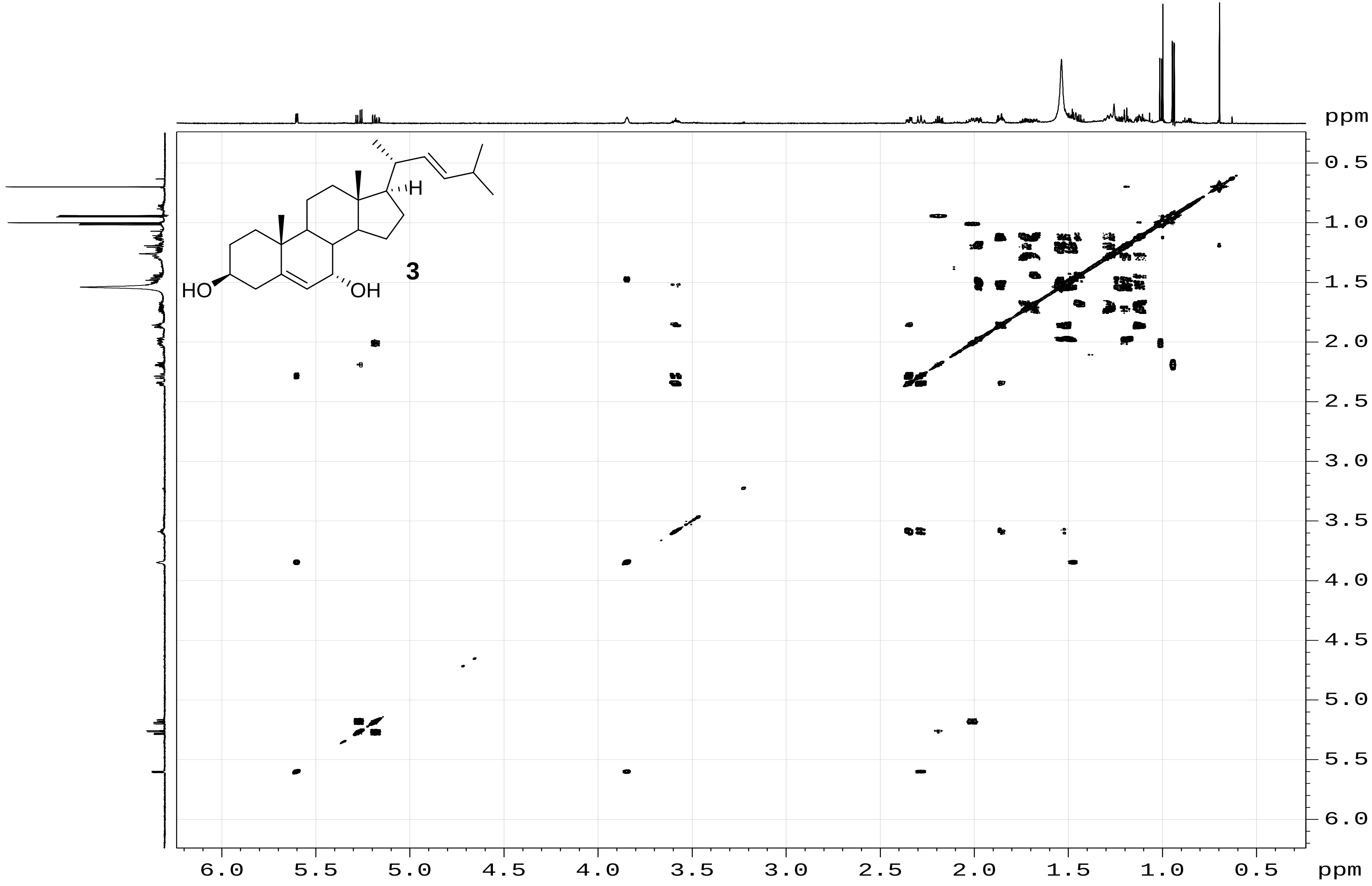

S16 ROESY NMR (700.13 MHz) spectrum of the (22*E*)-24-*nor*-cholesta-5,22-diene-3β,7α-diol (**3**) in $CDCl_3$

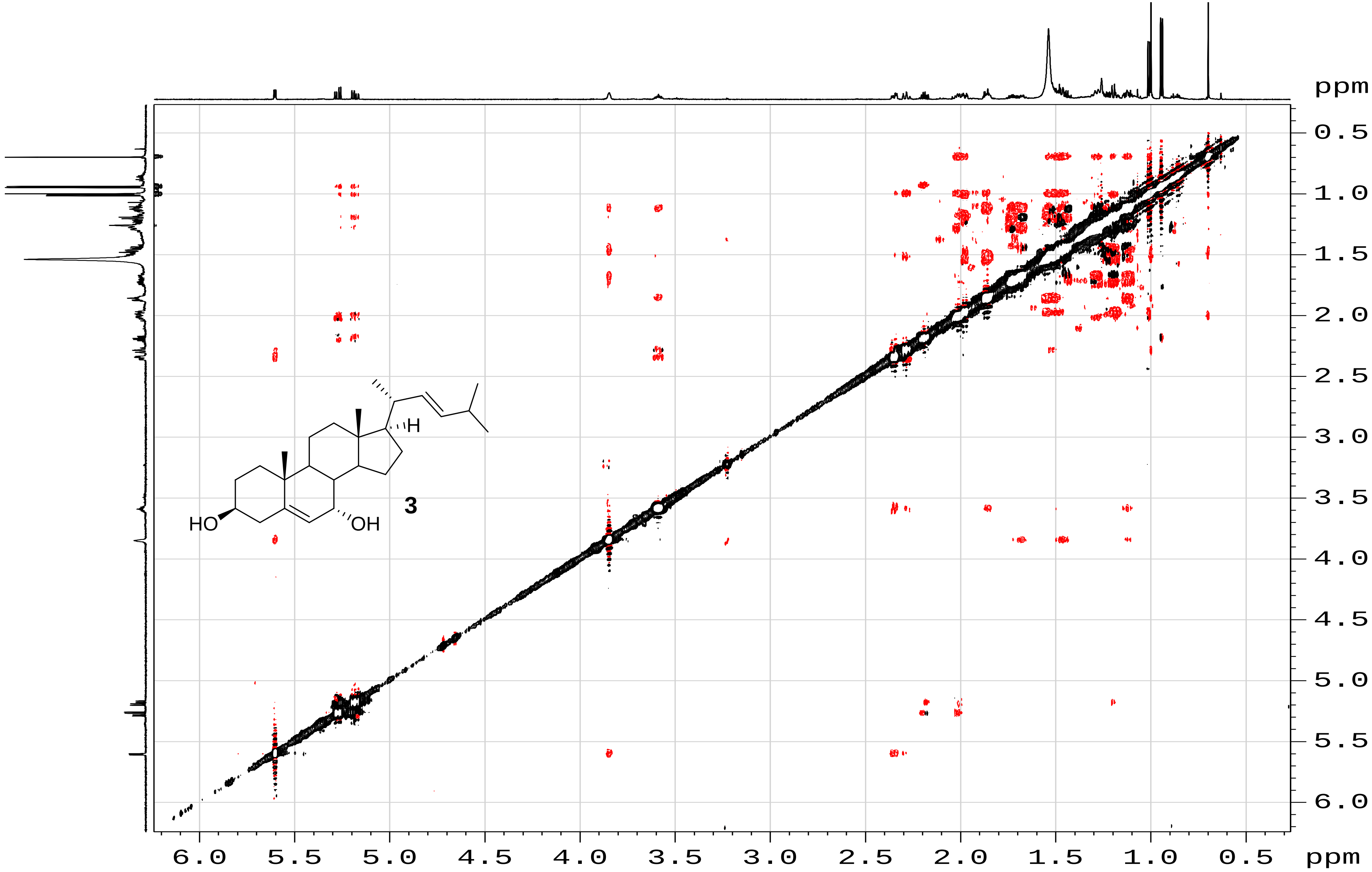

S17 HSQC NMR (700.13 MHz) spectrum of the (22*E*)-24-*nor*-cholesta-5,22-diene-3β,7α-diol (**3**) in $CDCl_3$

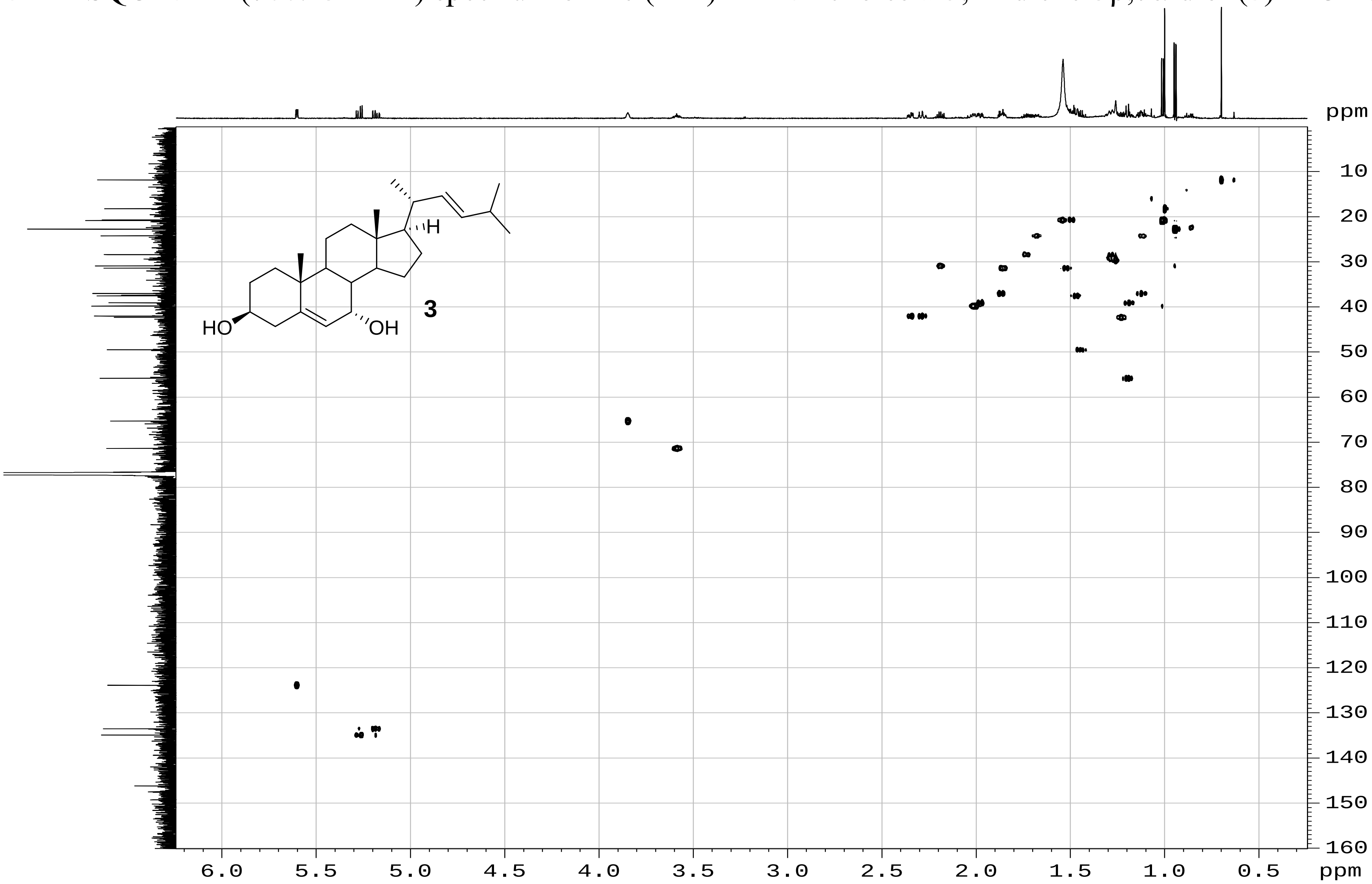

S18 HMBC NMR (700.13 MHz) spectrum of the (22*E*)-24-*nor*-cholesta-5,22-diene-3β,7α-diol (**3**) in $CDCl_3$

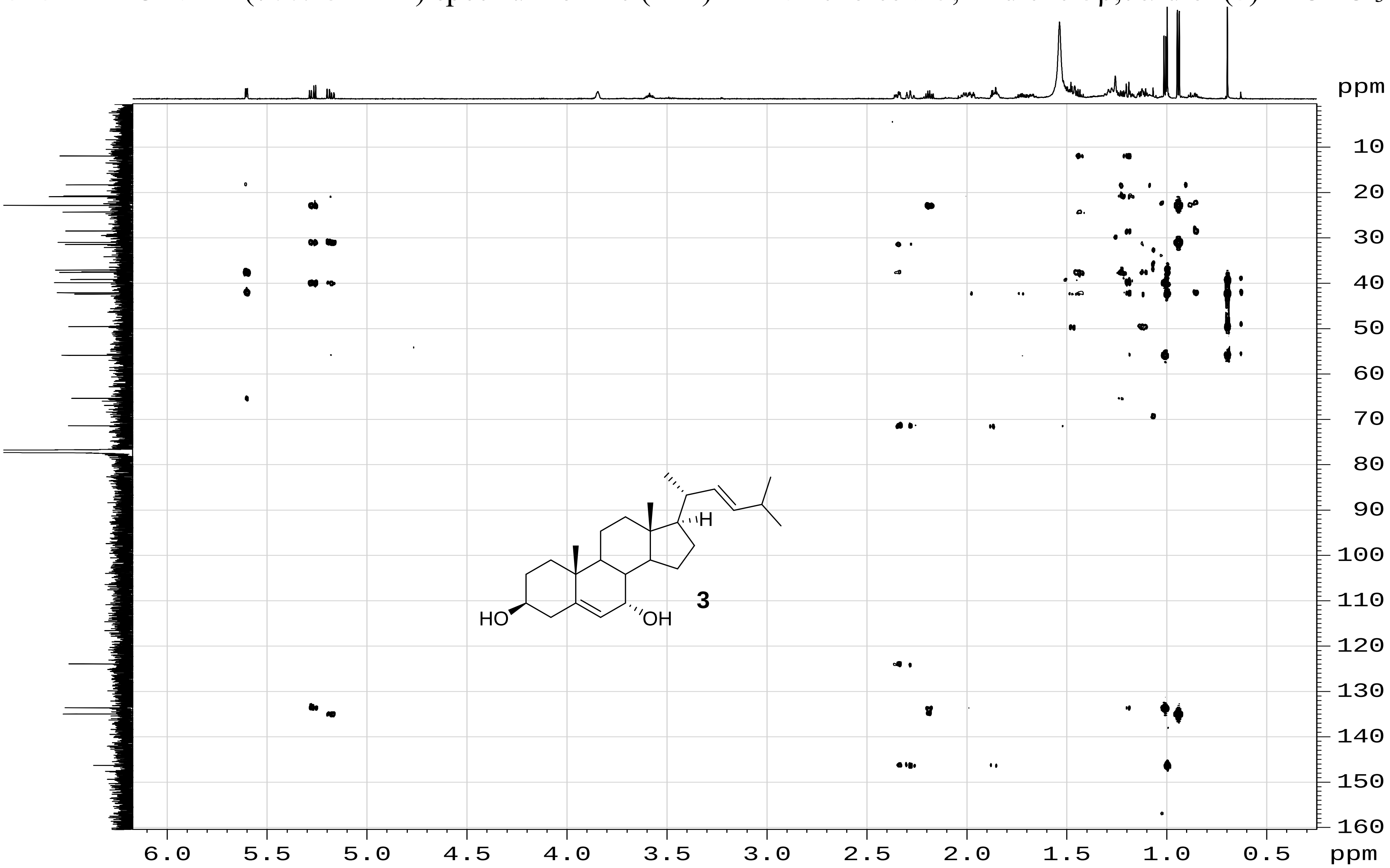

S19 ^{1}H NMR (500.13 MHz) spectrum of the (22*E*)-24-*nor*-cholesta-5,22-diene-3β,7β -diol (**4**) in $CDCl_3$

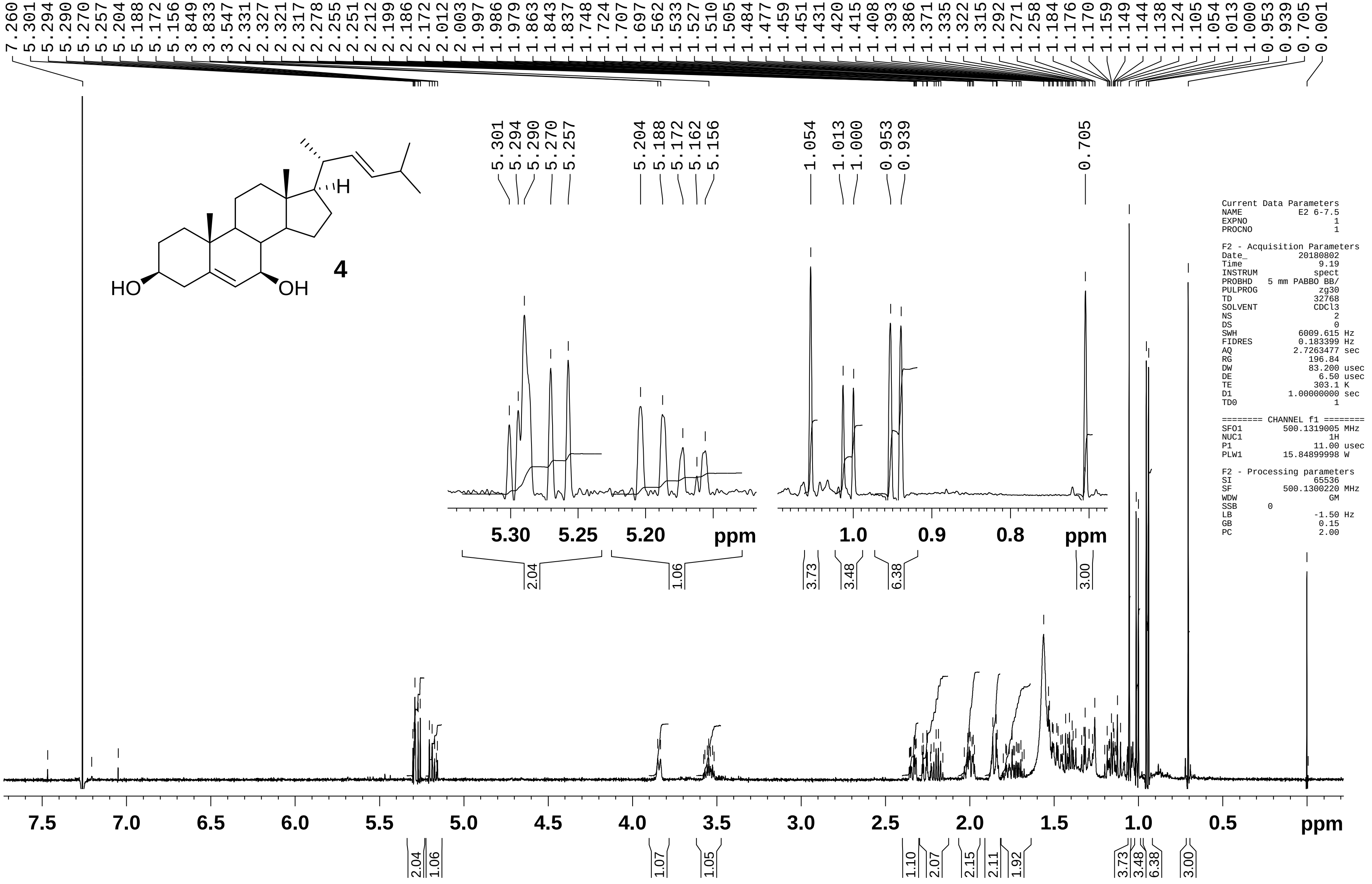

S20 ^{13}C NMR (125.76 MHz) spectrum of the (22*E*)-24-*nor*-cholesta-5,22-diene-3β,7β-diol (**4**) in $CDCl_3$

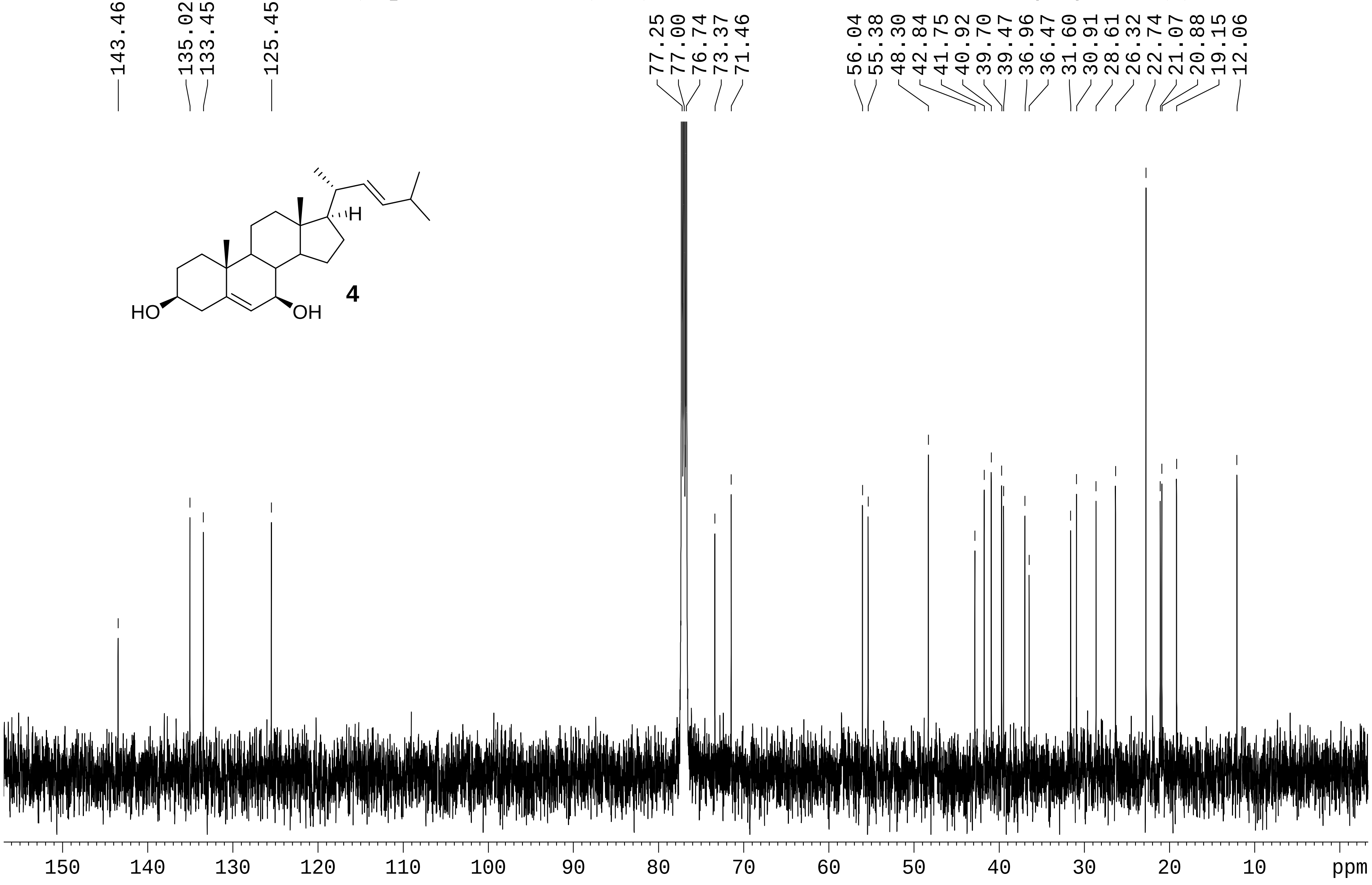

S21 COSY NMR (500.13 MHz) spectrum of the (22*E*)-24-*nor*-cholesta-5,22-diene-3β,7β-diol (**4**) in $CDCl_3$

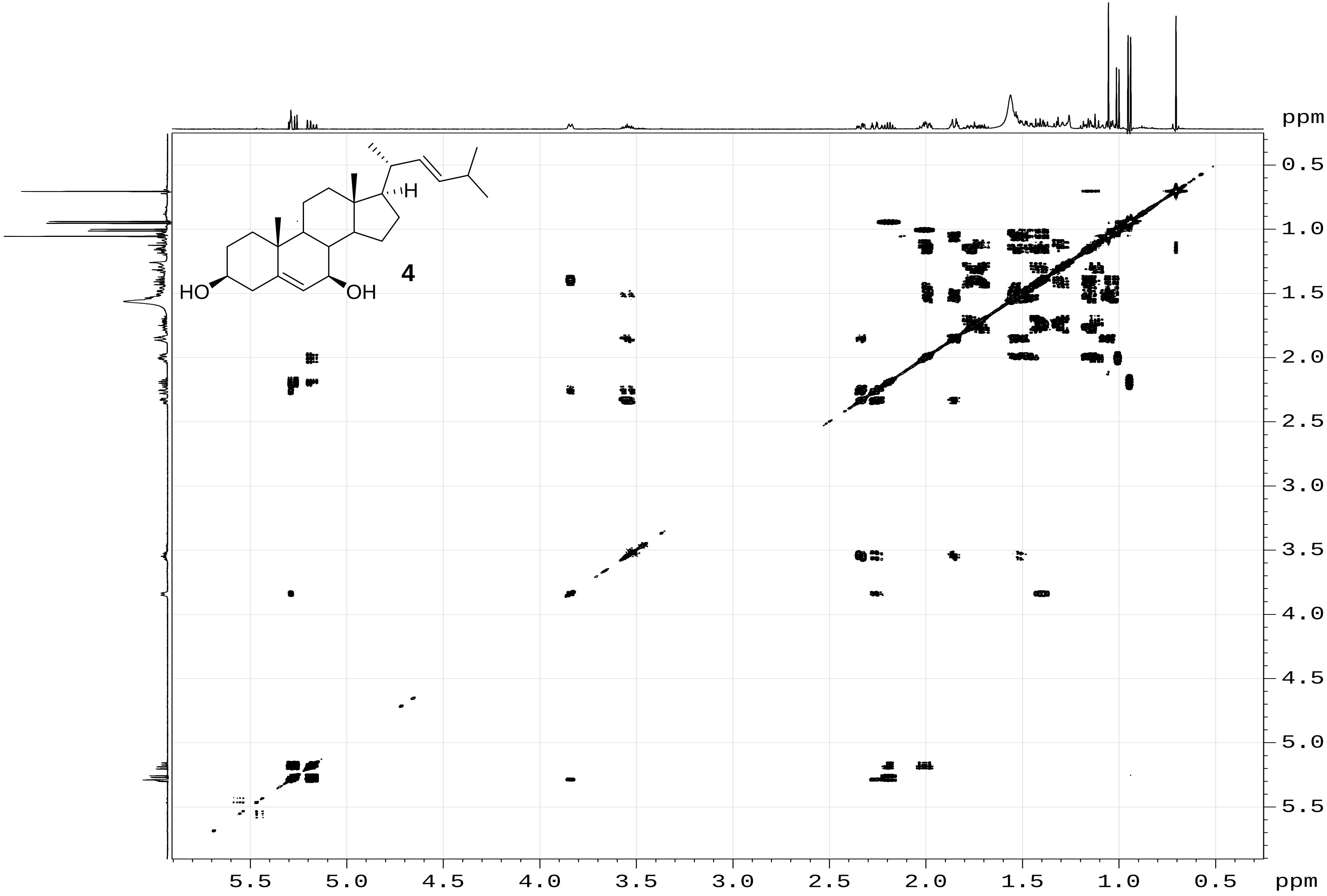

S22 ROESY NMR (500.13 MHz) spectrum of the (22*E*)-24-*nor*-cholesta-5,22-diene-3β,7β-diol (**4**) in $CDCl_3$

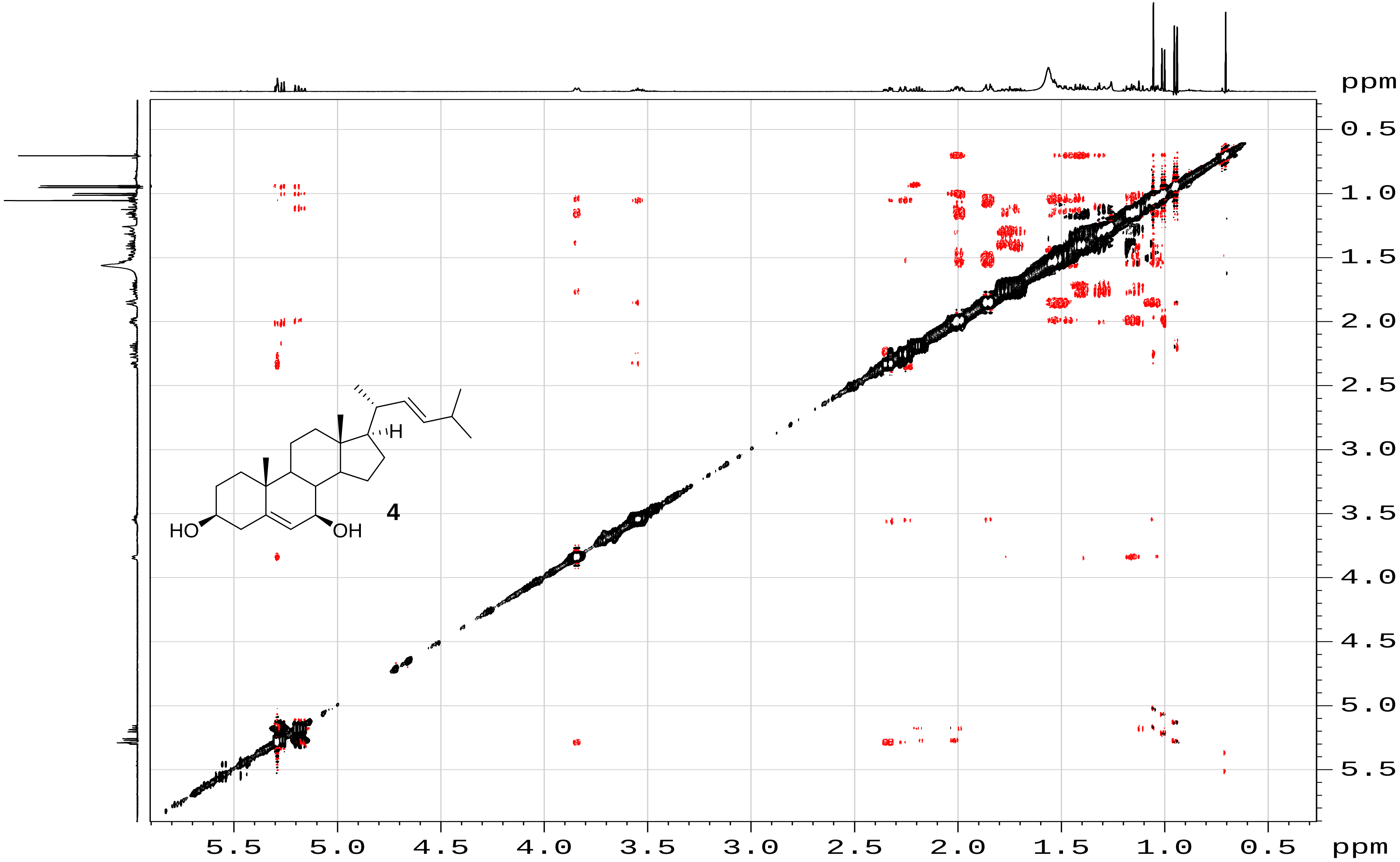

S23 HSQC NMR (500.13 MHz) spectrum of the (22*E*)-24-*nor*-cholesta-5,22-diene-3β,7β-diol (**4**) in $CDCl_3$

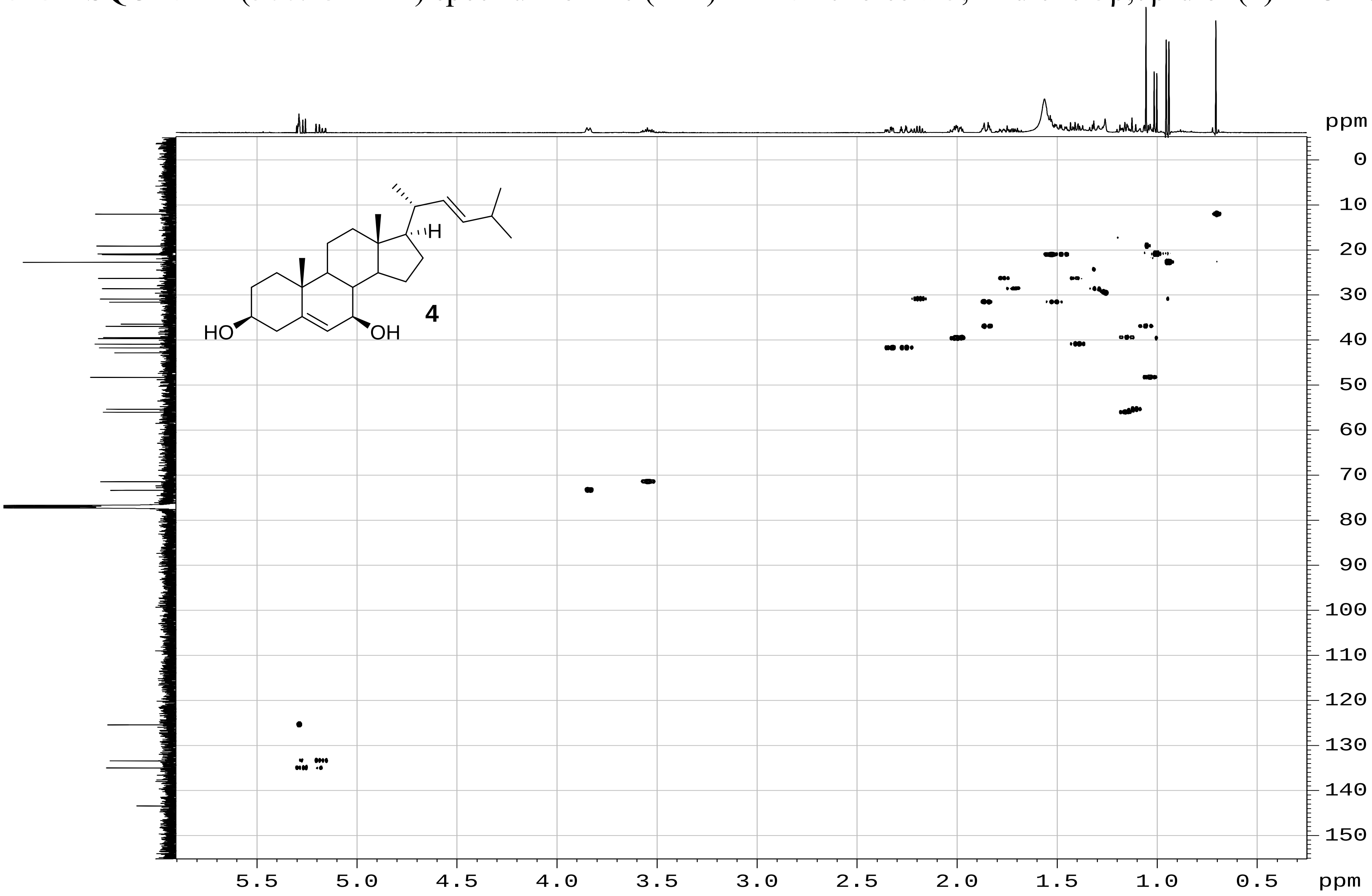

S24 HMBC NMR (500.13 MHz) spectrum of the (22*E*)-24-*nor*-cholesta-5,22-diene-3β,7β-diol (**4**) in $CDCl_3$

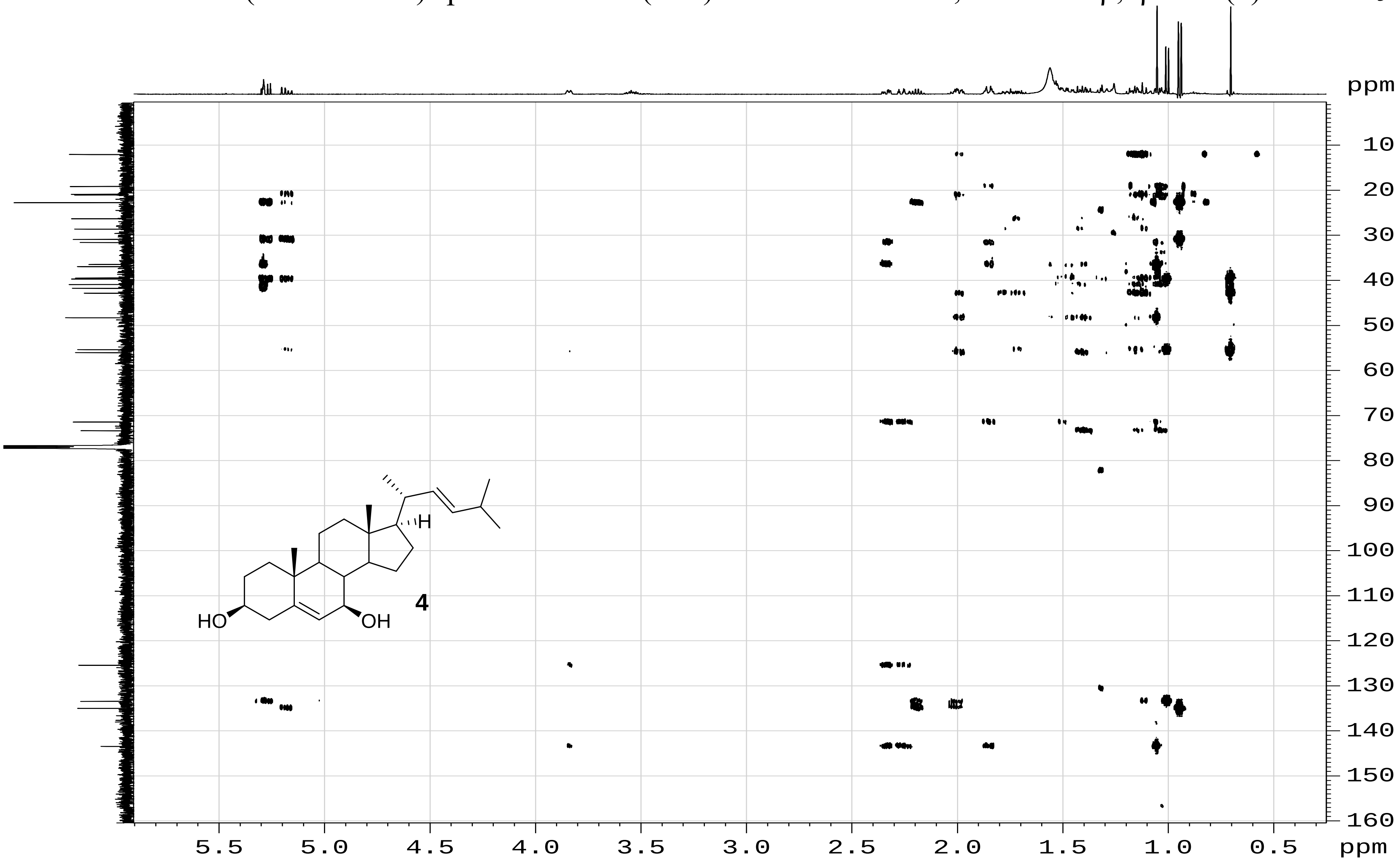

S25 Viability of Neuro2a cells

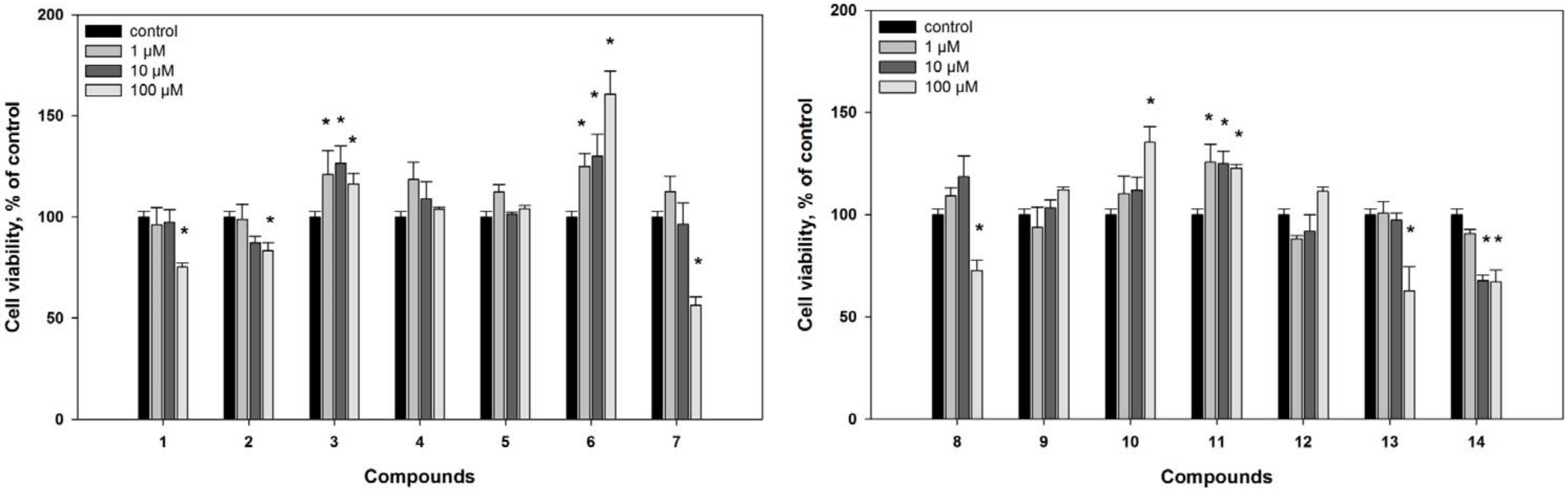

(a) (b)

Influence of compounds **1**-**7** (**a**) and **8**-**14** (**b**) on viability of Neuro2a cells by MTT assay.

* Statistically significant differences ($p \leq 0.05$) between results for control cells and cells incubated with compounds.

S26 ROS formation in Neuro2a cells

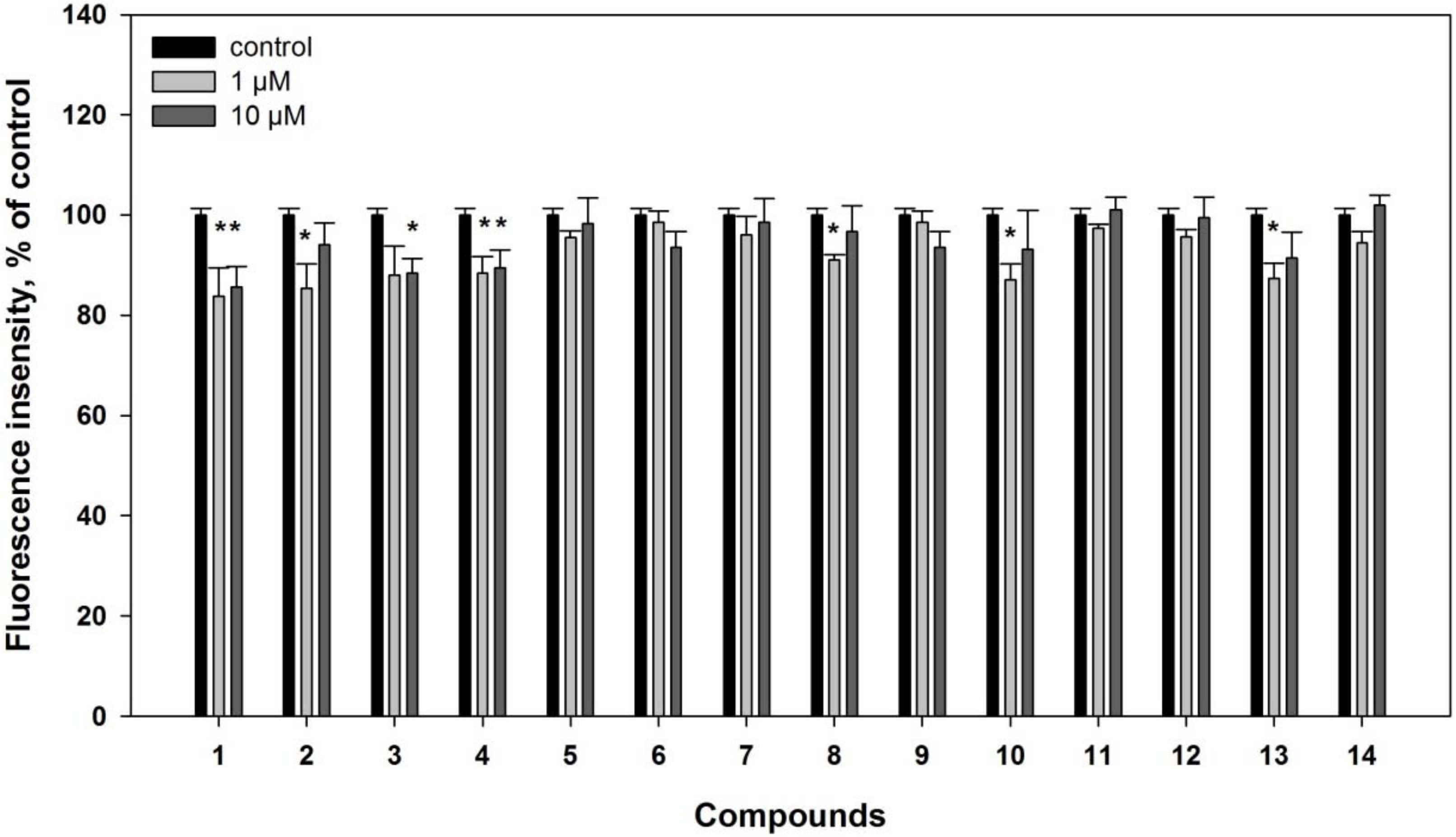

Influence of compounds **1**-**14** on ROS formation in Neuro2a cells.

*Statistically significant differences (*p*≤0.05) between results for control cells and cells incubated with compounds.

S27 Viability of Neuro2a cells treated with 6-OHDA

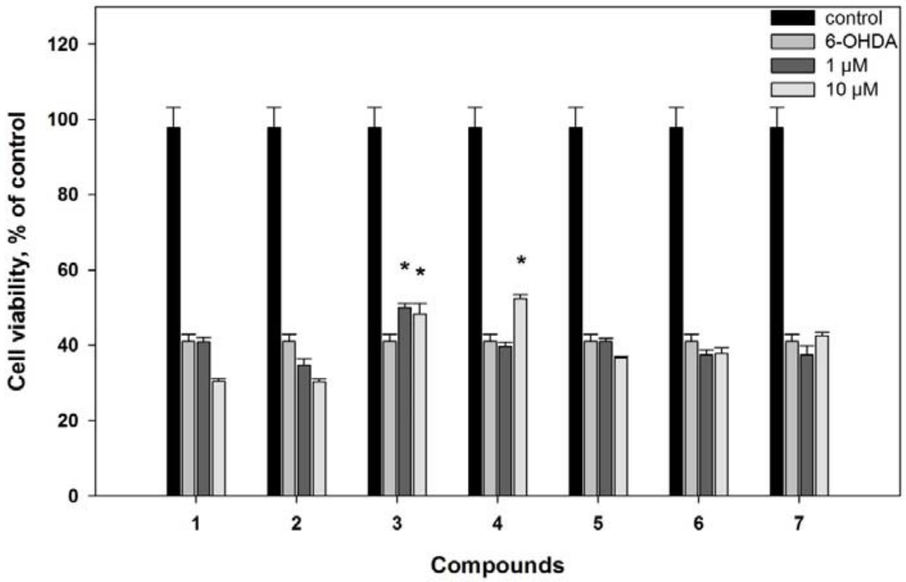

(a)

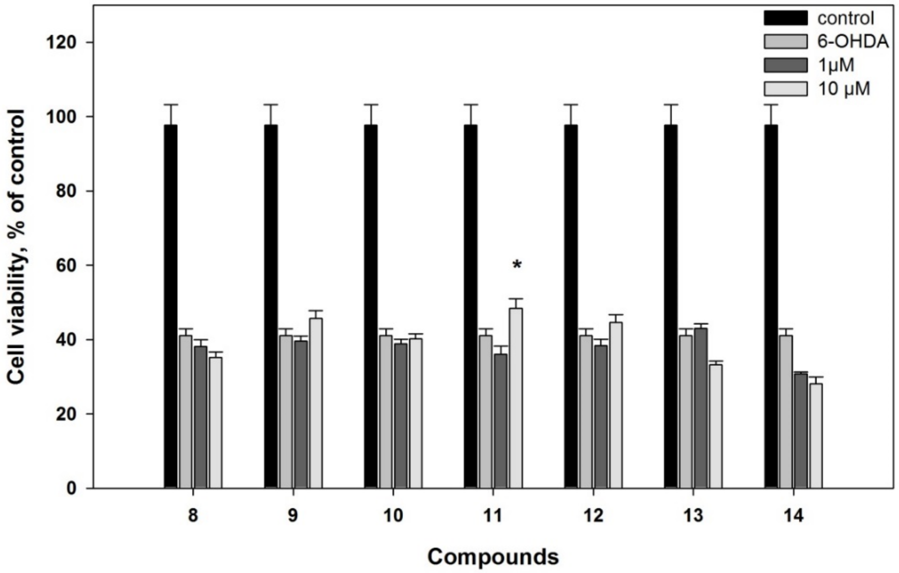

(b)

Influence of compounds **1**-**7** (**a**) and **8**-**14** (**b**) on viability of Neuro2a cells treated with 6-OHDA (50 μM).

* Statistically significant differences ($p \leq 0.05$) between results for 6-OHDA-treated cells and cells incubated with compounds.
